# Using deep learning to predict abdominal age from liver and pancreas magnetic resonance images

**DOI:** 10.1038/s41467-022-29525-9

**Published:** 2022-04-13

**Authors:** Alan Le Goallec, Samuel Diai, Sasha Collin, Jean-Baptiste Prost, Théo Vincent, Chirag J. Patel

**Affiliations:** 1 Department of Biomedical Informatics, Harvard Medical School, Boston, MA 02115 USA; 2 Department of Systems, Synthetic and Quantitative Biology, Harvard University, Cambridge, MA 02118 USA

**Keywords:** Predictive markers, Genetics research, Machine learning, Genome

## Abstract

With age, the prevalence of diseases such as fatty liver disease, cirrhosis, and type two diabetes increases. Approaches to both predict abdominal age and identify risk factors for accelerated abdominal age may ultimately lead to advances that will delay the onset of these diseases. We build an abdominal age predictor by training convolutional neural networks to predict abdominal age (or “AbdAge”) from 45,552 liver magnetic resonance images [MRIs] and 36,784 pancreas MRIs (R-Squared = 73.3 ± 0.6; mean absolute error = 2.94 ± 0.03 years). Attention maps show that the prediction is driven by both liver and pancreas anatomical features, and surrounding organs and tissue. Abdominal aging is a complex trait, partially heritable (h_g^2^ = 26.3 ± 1.9%), and associated with 16 genetic loci (e.g. in *PLEKHA1* and *EFEMP1*), biomarkers (e.g body impedance), clinical phenotypes (e.g, chest pain), diseases (e.g. hypertension), environmental (e.g smoking), and socioeconomic (e.g education, income) factors.

## Abstract (web summary)

Approaches to both determine abdominal age and identify risk factors for accelerated abdominal age will help delay the onset of several diseases. Here, the authors build an abdominal age predictor by training convolutional neural networks to predict abdominal age from liver and pancreas MRIs.

## Introduction

With age, different abdominal organs and tissues undergo important changes^1^. For example, the liver changes both at the cellular (e.g hepatocyte volume, polyploidy, accumulation of dense bodies, reduced smooth endoplasmic reticulum, reduced number of mitochondria) and the macroscopic (e.g reduced volume by 20–40%, up to 35% reduced blood flow) levels, becoming more vulnerable to age-related liver diseases such as liver fibrosis, non-alcoholic fatty liver disease, alcoholic liver disease, and hepatitis C^2,3^. Similarly, the pancreas undergoes fibrosis, atrophies, becomes fattier and vulnerable to age-related pancreas-diseases, leading to age related-pancreas disorders such as diabetes, cancer, gallstones and inflammatory pancreatic disease^4–6^. Other organs, such as the gastrointestinal tract, undergo similar processes^7^.

Biological age predictors can help understand the etiology of abdominal organ aging, with the hope to delay the onset of the aforementioned age-related diseases, and others. Biological age represents the state of the body of an individual and it is the true underlying cause of age-related diseases. It is in contrast with chronological age --commonly referred to as age-- the time since the individual’s birth. Biological age predictors are typically built by training machine learning models to predict chronological age. The prediction outputted by the model can then be interpreted as the individual’s biological age. Predictors have already been built on diverse organ datasets such as brain magnetic resonance images [MRIs]^8^, heart MRIs^9^, electrocardiograms^9,10^, carotid ultrasound images^11^, pulse wave analysis records^11^, full-body X-ray images^12,13^, chest X-ray images^14^, eye fundus images^15^, facial features^16^, blood samples^17^, DNA methylation^18^, transcriptomics^19^, proteomics^20^, microbiome^21–23^ and physical activity measurements^24^. However, to our knowledge, abdominal MRIs such as liver and pancreas MRIs have not been used to predict age.

**Fig. 1:**
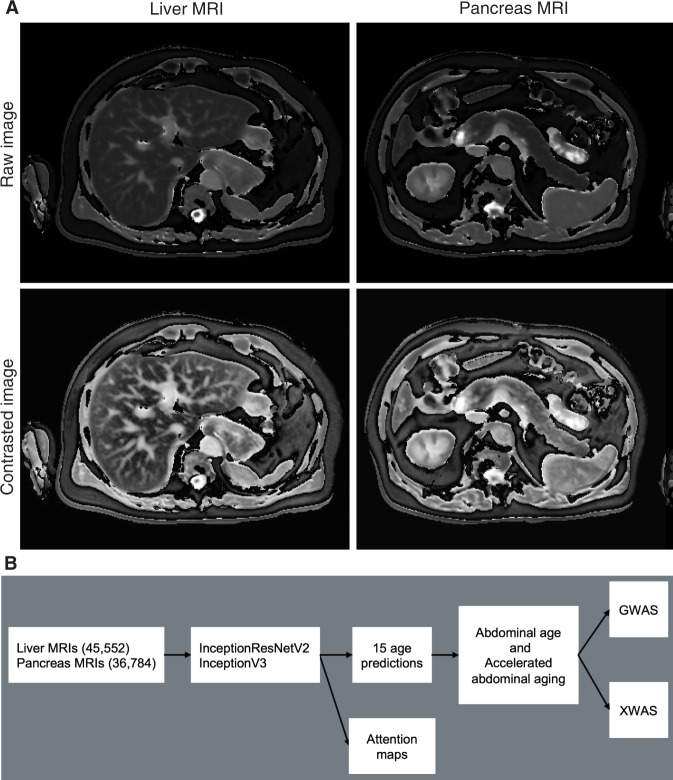
Overview of the datasets and analytic pipeline. **A** Sample liver and pancreas MRI images, both raw and preprocessed with a contrasting filter. **B** Schematic of Analytic pipeline. **B**: Sample sizes are in parentheses.

In the following, we built the first abdominal age predictor, called AbdAge. We leveraged 45,552 liver MRIs and 36,784 pancreas MRIs (Fig. 1A) collected from UK Biobank^25^ participants aged 37–82 year-old and trained deep convolutional neural networks to predict age from these datasets. We then performed a genome-wide association study [GWAS] to estimate the heritability of accelerated abdominal aging and to identify single nucleotide polymorphisms [SNPs] associated with this phenotype. Similarly, we performed an X-wide association study [XWAS] to identify biomarkers, clinical phenotypes, diseases, environmental and socioeconomic variables associated with accelerated abdominal aging. (Fig. 1B).

## Results

### Chronological age prediction

**Fig. 2:**
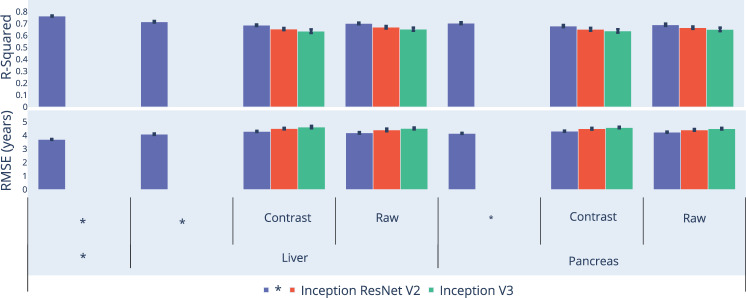
Prediction performance (R^2^ and RMSE) for AbdAge, Liver and Pancreas Age models. “Contrast” denotes contrasted image. *represent ensemble models. R2: R-squared of predicted versus actual age. RMSE: Root-mean-squared error. Error bars represent 2 SD of the bootstrapped estimate.

We leveraged the UK Biobank, a dataset containing 48,067 liver MRIs and 39,940 pancreas MRIs (Fig. 1A) collected from participants aged 37–82 years (Supplementary Fig. S1). After filtering out low quality images, we used deep convolutional neural networks and transfer learning to predict age from 45,552 liver MRIs (R-Squared [R^2^] = 71.5 ± 0.6%; mean absolute error [MAE] = 3.24 ± 0.04 years; root mean squared error [RMSE] = 4.1 ± .05 years) and from 36,784 pancreas MRIs (R^2^ = 70.3 ± 0.8; MAE = 3.30 ± 0.04 years; RMSE of 4.1 ± 0.04 years), which we then combined into an ensemble model that predicted age (or AbdAge) with a R^2^ of 76.3 ± 0.6, a MAE of 2.94 ± 0.03 years, and a RMSE of 3.7 ± 0.03. (Fig. 2).

We defined liver age as the prediction outputted by the liver MRIs-based model, pancreas age as the prediction outputted by the pancreas MRIs-based model, and abdominal age as the prediction outputted by the ensemble model leveraging both liver and pancreas MRIs. All predictions were corrected for the analytical bias in the age prediction residuals (see Methods).

### Identification of features driving abdominal age prediction

**Fig. 3:**
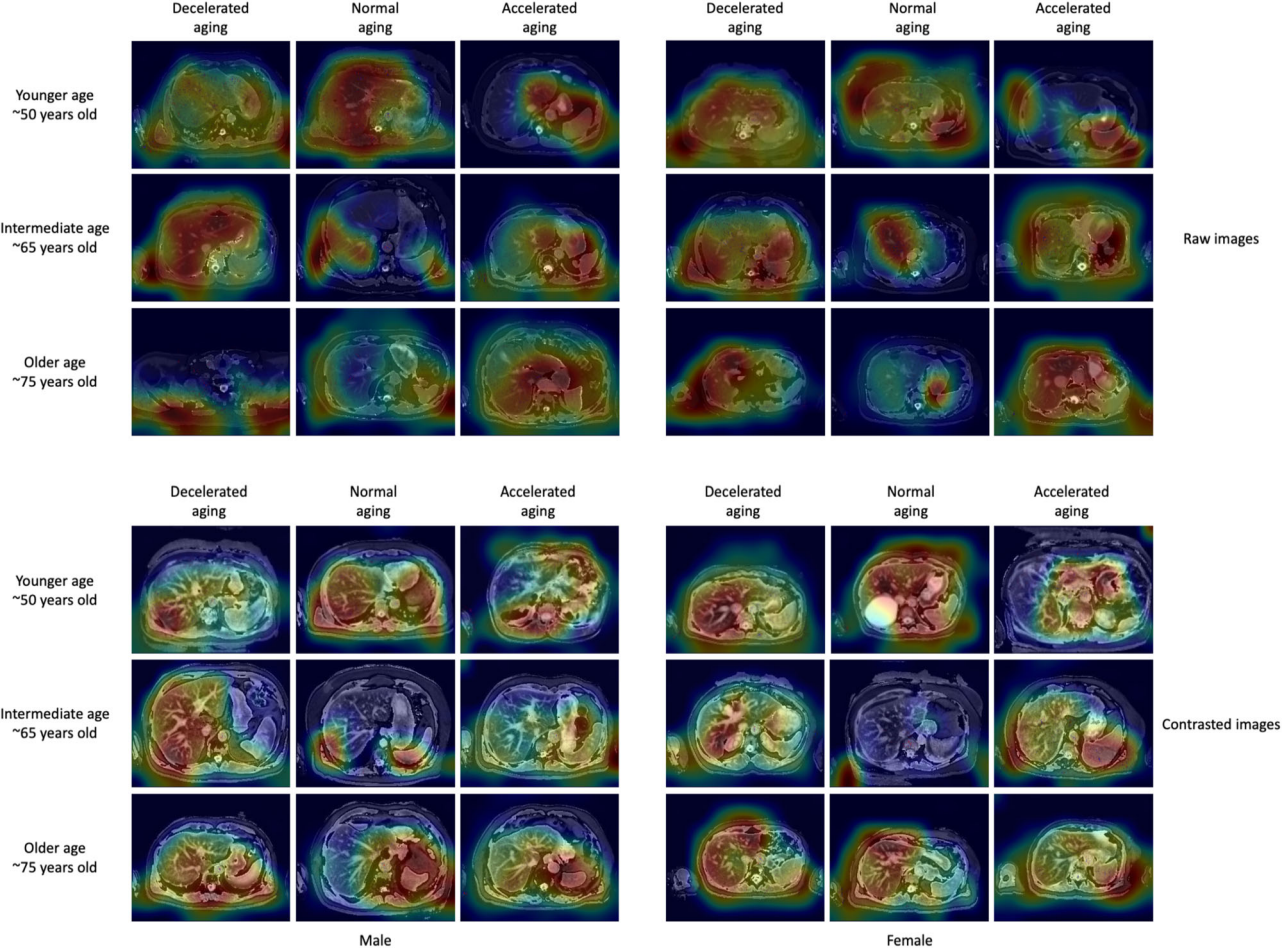
Sample attention maps for liver MRI-based models on raw and contrasted images. “Warm” filter colors (more red) highlight regions of high importance according to the Grad-RAM map. Actual chronological ages are obscured.

**Fig. 4:**
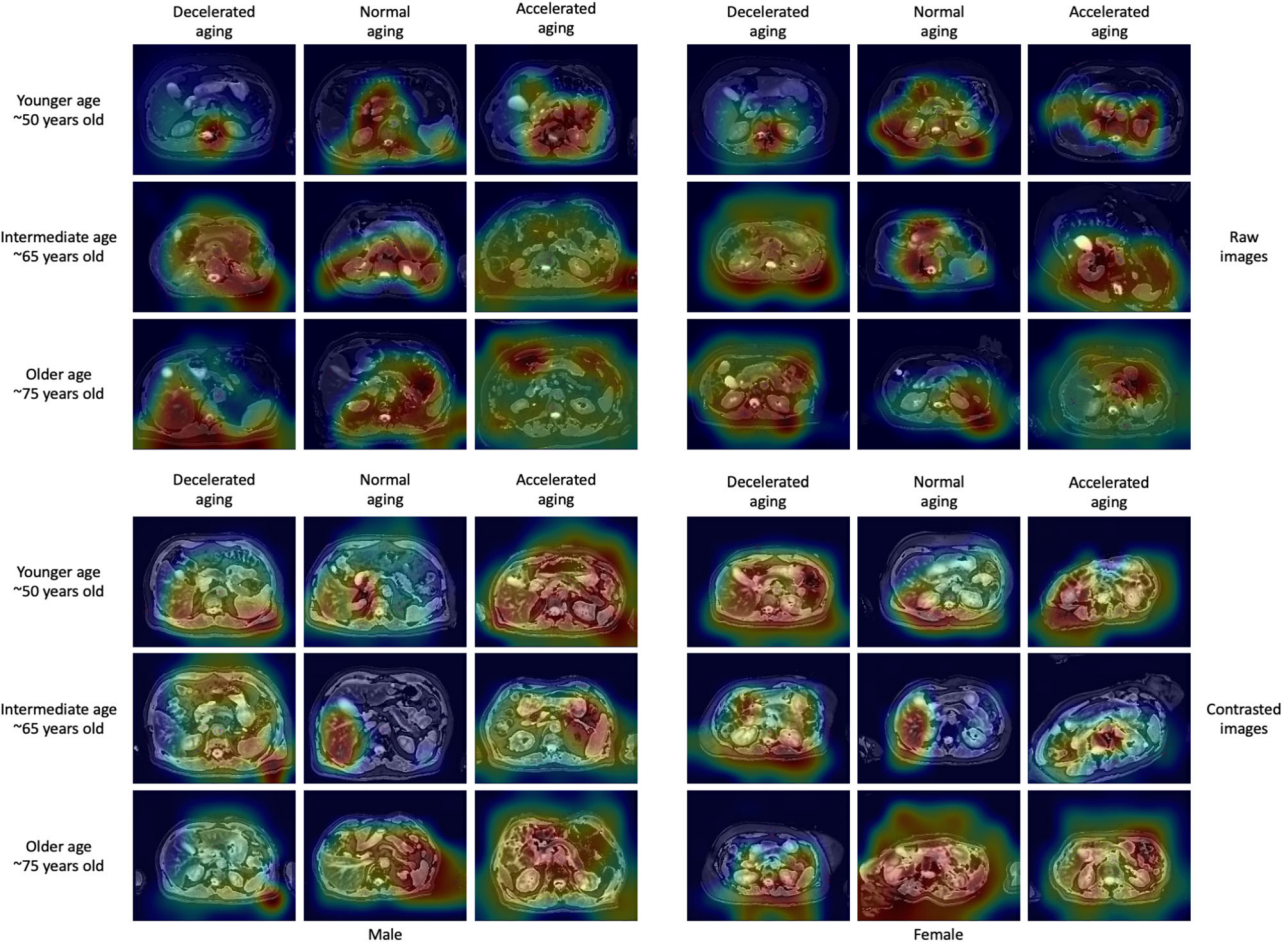
Sample attention maps for pancreas MRI-based models on raw and contrasted images. “Warm” filter colors (more red) highlight regions of high importance according to the Grad-RAM map. Actual chronological ages are obscured.

For liver MRI-based models, attention maps highlighted the liver along with other abdominal structures such as the stomach, the spleen, muscle, and adipose tissue (Fig. 3). Similarly, for pancreas MRI-based models, attention maps highlighted diverse abdominal regions across participants, including the liver (Fig. 4).

### Genetic factors and heritability of accelerated abdominal aging

We performed three genome wide association studies [GWASs] to estimate the GWAS-based heritability of abdominal (AbdAge, h_g_^2^ = 26.3 ± 1.9%), liver MRI-based (h_g_^2^ = 22.3 ± 1.5%), and pancreas MRI-based (h_g_^2^ = 22.1 ± 1.9%) accelerated aging. The distribution of the accelerated AbdAge, liver age, and pancreas age is seen in Supplementary Fig. S2. GWAS quality control is documented in the Supplementary Methods and Supplementary Figs. S3–S4.

**Fig. 5:**
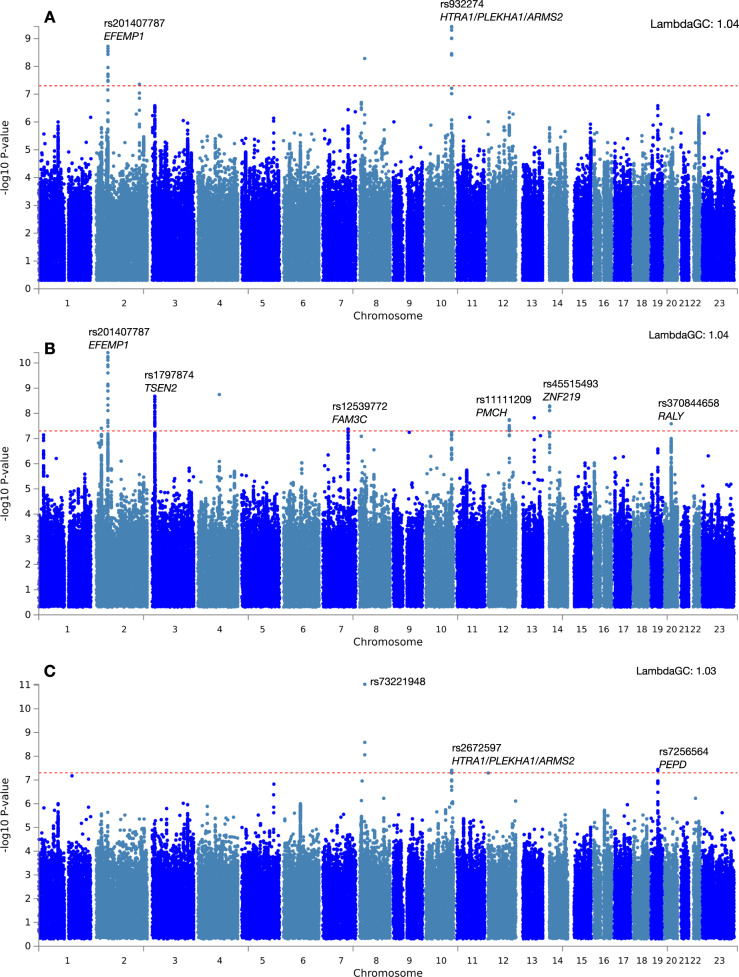
-log10(pvalue) vs. chromosomal position: GWAS on AbdAge, Liver, and Pancreas Accelerated Age. **A** GWAS results for accelerated abdominal aging (AbdAge); Lambda GC:1.04. Sample size: 32,475. **B** Liver Accelerated Age. Lambda GC: 1.04; Sample size: 40,760 and **C** Pancreas Accelerated Age. LambdaQC: 1.03; Sample Size: 32,548;-log10(*p*-value) vs. chromosomal position of locus. Dotted line denotes 5 × 10^−8^. All GWAS *p* values reported are 2-sided and not corrected as shown for multiple comparisons; however, all results lower than 5 × 10–8 achieved Bonferroni-level of significance.

We identified three, two, and eleven independent loci associated with Abdomen (AbdAge), Pancreas, and Liver Accelerated Age respectively (Table 1, Fig. 5, Supplementary Fig. S5, Supplementary Data Table S1). We found two different loci in AbdAge (rs932274, *p* = 1E−10) and Pancreas Accelerated Age (rs2672597, p = 3.9E−9) respectively that are close to genes implicated in age-related macular degeneration (*PLEKHA1*, *ARMS2, HTRA1*). We found a locus with lead SNP (rs201407787) in common with both AbdAge (*p* = 1E−9) and Liver Age (*p* = 1E−11) that maps to an intergenic region of *EFEMP1* (Supplementary Data Table S1).

**Table 1:** Genome-wide significant loci implicated in accelerated Abdomen Age (AbdAge), Pancreas Age, and Liver Age.

Age phenotype	rsID	Chr	Position	Alleles	MAF	Beta	SE	*P*-value	# SNPs	Closest genes
AbdAge	rs201407787	2	56071109	C;T	0.123	0.215	0.036	1.90E−09	20	*EFEMP1*
AbdAge	rs2216113	2	206436181	G;A	0.151	0.178	0.033	4.40E−08	5	*PARD3B*
AbdAge	rs932274	10	124225364	C;T	0.269	−0.175	0.028	3.70E−10	9	*PLEKHA1;ARMS2;HTRA1*
Pancreas Age	rs2672597	10	124226199	G;A	0.270	−0.165	0.030	3.90E−08	9	*PLEKHA1;ARMS2;HTRA1*
Pancreas Age	rs7256564	19	33889593	A;G	0.308	0.155	0.028	3.60E−08	17	*PEPD*
Liver Age	rs552571374	2	25148623	G;C	0.101	−0.202	0.037	3.90E−08	41	*ADCY3;DNAJC27;EFR3B*
Liver Age	rs201407787	2	56071109	C;T	0.123	0.225	0.034	3.90E−11	20	*EFEMP1*
Liver Age	rs3791675	2	56111309	C;T	0.231	−0.160	0.027	4.70E−09	53	
Liver Age	rs1797874	3	12529592	C;A	0.441	−0.140	0.023	2.10E−09	123	*TSEN2;C3orf83;MKRN2*
Liver Age	rs13107325	4	103188709	C;T	0.080	0.271	0.045	1.80E−09	5	*BANK1;SLC39A8*
Liver Age	rs12539772	7	121005636	T;A	0.260	−0.141	0.026	4.20E−08	23	*WNT16;FAM3C*
Liver Age	rs11111209	12	102600598	T;C	0.114	0.201	0.036	1.80E−08	14	
Liver Age	rs77353655	12	102671553	A;G	0.107	0.201	0.037	4.90E−08	13	
Liver Age	rs76652635	13	74689496	A;G	0.072	−0.257	0.045	1.50E−08	2	*KLF12*
Liver Age	rs45515493	14	21572642	C;G	0.118	−0.195	0.033	5.10E−09	14	*NDRG2;ARHGEF40;ZNF219;TMEM253*
Liver Age	rs370844658	20	32679575	A;ATT	0.341	−0.133	0.024	2.60E−08	213	*RALY;EIF2S2*

*Chr*: chromosome number, position: position on the chromosome, *Alleles*: effect;non-effect allele, *MAF*: minor allele frequency, *Beta*: beta coefficient of GWAS, *SE*: standard error of beta coefficient, *P*-value: *p*-value on beta coefficient, *#SNPS*: number of SNPs in LD with main SNP, *Closest Genes*: closest genes to rsID. All *p*-values are two sided and not corrected for multiple comparisons, but reported findings are GWA-significant (5 × 10^−8^).

### Biomarkers, clinical phenotypes, diseases, environmental and socioeconomic variables associated with accelerated abdominal aging

We use “X” to refer to all nongenetic variables measured in the UK Biobank (biomarkers, clinical phenotypes, diseases, family history, environmental and socioeconomic variables). We performed an X-Wide Association Study [XWAS] to identify which of the 4372 biomarkers classified in 21 subcategories (Supplementary Data Table S3), 187 clinical phenotypes classified in 11 subcategories (Supplementary Data Table S5), 2073 diseases classified in 26 subcategories (Supplementary Data Table S8), 92 family history variables (Supplementary Data Table S11), 265 environmental variables classified in nine categories (Supplementary Data Table S14), and 91 socioeconomic variables classified in five categories (Supplementary Data Table S17) are associated (p-value threshold of 0.05 and Bonferroni corrected) with accelerated abdominal aging in the different dimensions. We summarize our findings for general accelerated abdominal aging below. Please refer to the supplementary data tables (Supplementary Data Table S5, Supplementary Data Table S4, Supplementary Data Table S6, Supplementary Data Table S7, Supplementary Data Table S9, Supplementary Data Table S10, Supplementary Data Table S15, Supplementary Data Table S16, Supplementary Data Table S18, Supplementary Data Table S19) for a summary of non-genetic factors associated with general, liver MRI-based and pancreas MRI-based accelerated abdominal aging. The exhaustive results can be found in Supplementary Data Table S20 and explored at https://www.multidimensionality-of-aging.net/xwas/univariate_associations.

Out of the 17,459 associations tested, 1456 (8.34%) were significant, with an average absolute value of 0.044 (range: 0.022−0.091; IQR: .034−0.053). In the below, we describe some of the top-ranking correlations.

#### Biomarkers associated with accelerated abdominal aging

The three biomarker categories most associated with accelerated abdominal aging are body impedance, blood pressure, and pulse wave analysis. Specifically, 100.0% of impedance biomarkers are associated with accelerated abdominal aging, with the three largest associations being with right arm impedance (correlation = 0.056), left arm impedance (correlation = 0.055), and whole body impedance (correlation = 0.042). 66.7% of blood pressure biomarkers are associated with accelerated abdominal aging, with the two associations being with diastolic blood pressure (correlation = 0.050) and systolic blood pressure (correlation = 0.036). 46.7% of pulse wave analysis biomarkers are associated with accelerated abdominal aging, with the three largest associations being with diastolic blood pressure (correlation = 0.050), systolic blood pressure (correlation = 0.048), and mean arterial pressure (correlation = 0.046).

Conversely, the three biomarker categories most associated with decelerated abdominal aging are hand grip strength, cognitive symbol digit substitution, and bone heel densitometry. Specifically, 100% of hand grip strength biomarkers are associated with decelerated abdominal aging, with the two associations being with left and right hand grip strengths (respective correlations of 0.056 and 0.049). 100.0% of symbol digit substitution (a cognitive test) biomarkers are associated with decelerated abdominal aging, with the two associations being with the number of symbol digit matches made correctly (correlation = 0.036) and the number of symbol digit matches attempted (correlation = 0.035). 83.3% of heel bone densitometry biomarkers are associated with decelerated abdominal aging, with the three largest associations being with heel quantitative ultrasound index (correlation = 0.091), heel bone mineral density (correlation = 0.090), and speed of sound through heel (correlation = 0.089). In addition, we observed smaller correlations between blood, anthropometry, and biochemical variables (Supplementary Figure S6).

#### Clinical phenotypes associated with accelerated abdominal aging

The three clinical phenotype categories most associated with accelerated abdominal aging are general health, chest pain, and breathing. Specifically, 50.0% of general health phenotypes are associated with accelerated abdominal aging, with the three largest associations being with overall health rating (correlation = 0.069), weight loss in the last year (correlation = 0.065), and long-standing illness, disability, or infirmity (correlation = 0.050). 50.0% of chest pain phenotypes are associated with accelerated abdominal aging, with the two associations being with chest pain or discomfort walking normally (correlation = 0.032) and chest pain due to walking ceasing when standing still (correlation = 0.023). 50.0% of breathing phenotypes are associated with accelerated abdominal aging (one association: shortness of breath walking on level ground; correlation = 0.031).

Conversely, the two clinical phenotype categories associated with decelerated abdominal aging are sexual factors (age first had sexual intercourse; correlation = 0.030) and general health (gained weight or no weight change in the last year, respective correlations of 0.032 and 0.024).

#### Diseases associated with accelerated abdominal aging

The three disease categories most associated with accelerated abdominal aging are cardiovascular diseases, general health, and pulmonary diseases. Specifically, 6.5% of cardiovascular diseases are associated with accelerated abdominal aging, with the three largest associations being with hypertension (correlation = 0.058), atrial fibrillation and flutter (correlation = 0.045), and chronic ischaemic heart disease (correlation = 0.029). 6.0% of general health variables are associated with accelerated abdominal aging, with the three largest associations being with personal history of disease (correlation = 0.046), personal history of medical treatment (correlation = 0.042), and receiving medical care (correlation = 0.030). 4.8% of pulmonary diseases are associated with accelerated abdominal aging, with the three largest associations being with chronic obstructive pulmonary disease (correlation = 0.034), asthma (correlation = 0.026), and pleural effusion (correlation = 0.024).

#### Environmental variables associated with accelerated abdominal aging

The three environmental variable categories most associated with accelerated abdominal aging are smoking, sun exposure and alcohol intake. Specifically, 37.5% of smoking variables are associated with accelerated abdominal aging, with the three largest associations being with pack years adult smoking as proportion of lifespan exposed to smoking (correlation = 0.090), pack years of smoking (correlation = .086), and past tobacco smoking: smoked on most or all days (correlation = 0.066). 20.0% of sun exposure variables are associated with accelerated abdominal aging, with the three largest associations being with facial aging: about your age (correlation = 0.039), facial aging: do not know (correlation = 0.038), and time spent outdoors in summer (correlation = 0.036). 17.2% of alcohol intake variables are associated with accelerated abdominal aging, with the three largest associations being with red wine intake (correlation = 0.043), champagne plus white wine intake (correlation = 0.043), and beer plus cider intake (correlation = 0.042).

Conversely, the three environmental variable categories most associated with decelerated abdominal aging are physical activity, smoking and diet. Specifically, 34.3% of physical activity variables are associated with decelerated abdominal aging, with the three largest associations being with practicing strenuous sports (correlation = 0.078), frequency of strenuous sports in the last four weeks (correlation = 0.077), and duration of strenuous sports (correlation = 0.076). 29.2% of smoking variables are associated with decelerated abdominal aging, with the three largest associations being with smoking status: never (correlation = 0.073), time from waking to first cigarette (correlation = 0.063), and age started smoking (correlation = 0.062). 7.0% of diet variables are associated with decelerated abdominal aging, with the three largest associations being with cereal intake (correlation = 0.058), no major dietary changes in the five years (correlation = 0.036), and bread intake (correlation = 0.030).

#### Socioeconomic variables associated with accelerated abdominal aging

The two socioeconomic variable categories that are associated with accelerated abdominal aging are social support (no leisure or social activity among the ones listed: correlation = 0.033) and household (renting from local authority, local council, or housing association: correlation = 0.028).

Conversely, the three socioeconomic variable categories most associated with decelerated abdominal aging are sociodemographics, employment, and education. Specifically, 14.3% of sociodemographics variables are associated with decelerated abdominal aging (one association: not receiving attendance/disability/mobility allowance. correlation = 0.040). 13.0% of employment variables are associated with decelerated abdominal aging, with the three largest associations being with length of working week for main job (correlation = 0.044), current employment status: in paid employment or self-employed (correlation = 0.043), and frequency of travelling from home to job workplace (correlation = 0.029). 12.5% of education variables are associated with decelerated abdominal aging (one association: college or university degree; correlation = 0.048).

### Predicting accelerated abdominal aging from biomarkers, clinical phenotypes, diseases, environmental variables, and socioeconomic variables

We predicted accelerated abdominal aging using variables from the different X-datasets categories (biomarkers, clinical phenotypes, diseases, environmental variables and socioeconomic variables). Specifically we built a model using the variables from each of their respective subcategories (e.g blood pressure biomarkers), and found that no modalities could explain more than 5% of the variance in accelerated abdominal aging.

### Phenotypic, genetic, and environmental correlation between liver MRI-based and pancreas MRI-based accelerated abdominal aging

Liver MRI-based and pancreas MRI-based accelerated abdominal aging are phenotypically correlated (0.526 ± 0.005). For comparison, the ensemble models trained on two datasets that differ only in their preprocessing (raw vs. contrasted images) yielded accelerated abdominal aging definitions that are 0.810 ± 0.001 correlated (liver MRIs) and 0.841 ± 0.002 correlated (pancreas MRIs). Liver MRI-based and pancreas MRI-based accelerated abdominal aging share genetic architecture, and are genetically 0.863 ± 0.036 correlated.

We found moderate to modest, but non-zero, Pearson correlation between AbdAge and other organs, ranging from 0.45 (Heart MRI-based biological age), 0.35 for musculoskeletal aging, to a low of 0.04 (OCT eye age) (Supplementary Figure S7), with a median correlation of 0.15. We found we had evidence for shared genetic architecture between AbdAge and Liver and Pancreas Accelerated Age (genetic correlation of 0.95). Further, we found a significant, but moderate, genetic correlation between AbdAge and accelerated musculoskeletal spinal aging (genetic correlation of 0.56) and low genetic correlation with skeletal knee and hip aging (genetic correlation of 0.23 and 0.25).

**Fig. 6:**
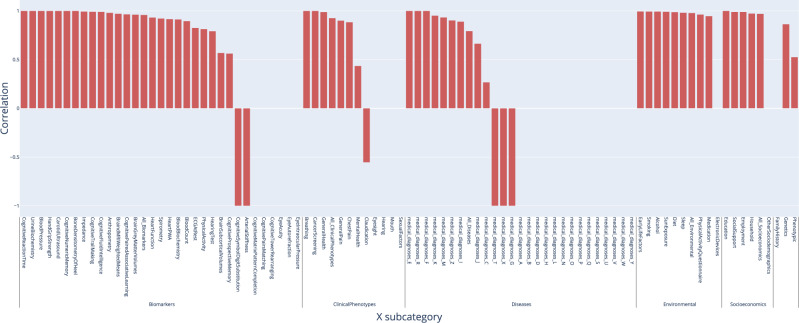
Correlation, or overlap, of non-genetic associations between Liver and Pancreas Aging. X-Axis denotes the category of non-genetic variable, and the y-axis is the correlation of the association sizes between Pancreas and Liver Aging phenotypes.

We also evaluated the correlation between liver MRI-based and pancreas MRI-based accelerated aging phenotypes in terms of their association with non-genetic variables. For example, are the environmental exposures associated with liver MRI-based accelerated aging similar to those associated with pancreas MRI-based accelerated aging? We found that the correlation between these two phenotypes to be 0.959 in terms of biomarkers, 0.926 in terms of associated clinical phenotypes, 0.793 in terms of diseases, 0.978 in terms of environmental variables and 0.969 in terms of socioeconomic variables (Fig. 6). These results can be interactively explored at https://www.multidimensionality-of-aging.net/correlation_between_aging_dimensions/xwas_univariate.

## Discussion

We built the first abdominal age predictor, AbdAge, by training deep convolutional neural networks to predict age from liver and pancreas MRI images (R^2^ = 73.3 ± 0.6; RMSE = 2.94 ± 0.03).

The attention maps of the models built on liver MRI images highlighted various abdominal regions including the liver, the stomach, the spleen, as well as muscle, bones, and adipose tissue. The attention maps of the models built on pancreas MRI images highlighted similar features aside from the pancreas, including the liver. The similarities between liver-based and pancreas-based attention maps suggest that our models do not capture liver aging and pancreas aging specifically, but instead capture general abdominal aging. The abdomen undergoes significant macroscopic changes as we age^1^, which were likely leveraged by the convolutional neural networks. In terms of liver aging, it is known that liver function decreases with age^26^ and that the liver ages at the cellular level, which is for example associated with low-grade inflammation^27^. There is less evidence that the liver undergoes clear macroscopic changes that could be captured by MRI images^28^, but it has been reported that with age the color of the liver gets darker, blood flow decreases, liver volume decreases^3,29^, and the prevalence of liver diseases, such as nonalcoholic fatty liver disease, alcoholic liver disease, cirrhosis, and fibrosis, increase with age^2^, which might have been leveraged by our models to predict chronological age^30^. In terms of pancreas aging, age-related changes visible on MRI images include pancreatic atrophy, fatty degeneration, and lobulation^31^. Finally, aging is also associated with abdominal changes in adipose tissue^32,33^, muscles^34–36^, and bones^37^.

Further confirming the intuition derived from the attention maps, liverMRI-based, and pancreas MRI-based accelerated aging are phenotypically, genetically, and environmentally correlated (respective correlations of .526, .863, and .978). As a consequence, the liver MRI-based age abdominal predictor should not be interpreted as a liver age predictor (nor should the pancreas MRI-based abdominal age predictor be considered specific to the pancreas). To build such organ-specific predictors, we believe it is necessary to perform image segmentation to pre-isolate the liver and pancreas features from their surrounding tissues and organs. Despite this limitation, liver and pancreas images did capture non-redundant/overlapping information regarding abdominal aging, as demonstrated by (1) the gain of prediction accuracy when combining both models (R^2^ = 73.3 ± 0.6 vs. 71.5 ± 0.6%) and (2) by the individual differences in GWAS signals (despite their large genetic correlation). Specifically, the ensemble model highlighted *EFEMP1* as associated with general abdominal aging, but this association was not found for pancreas MRI-based accelerated aging, despite analyzing sample sizes for the analysis (32,475 vs. 32,548). This difference, along with the fact that *EFEMP1* was also associated with liver MRI-based accelerated aging, suggests that this association is driven by features observable on liver MRIs and not on pancreas MRI.

The association between abdominal aging and blood biochemistry biomarkers such as alanine aminotransferase, aspartate aminotransferase suggest that abdominal aging is linked to liver function. Since age prediction is in part driven by the tissue surrounding the organs, a natural hypothesis is that the model also relies on body/liver fat percentage, whichi increases with age^38^. This hypothesis is partly supported by the fact that the biomarker category most associated with accelerated aging is body impedance, which increases with body fat percentage. Similarly, metabolism biomarkers such as HDL cholesterol, apolipoprotein A and glycated haemoglobin A1c (a diabetes biomarker) are associated with accelerated abdominal aging. However, and perhaps surprisingly, both body mass index, hip circumference, and weight are associated with decelerated abdominal aging. A possible explanation is that both old age^39^ and disease (e.g pancreas cancer^40^, cirrhosis^41^) are associated with weight loss.

Aside from these biomarkers which can be linked to abdominal health, accelerated abdominal aging is also associated with biomarkers, clinical phenotypes, and diseases linked to other organ systems’ health that cannot be not directly observed from liver and pancreas MRIs. For example, it is associated with poor cardiovascular health (e.g blood pressure, chest pain, hypertension, atrial fibrillation and flutter, chronic ischaemic heart disease), brain health (cognitive tests, brain MRI volumes, mental health disorders such as fed up feelings and mood swings), and pulmonary function (e.g spirometry, shortness of breath, chronic obstructive pulmonary disease, asthma, and pleural effusion). More generally, accelerated abdominal aging is associated with poor general health (e.g general health rating, recent weight loss, long-standing illness, disability or infirmity, personal history of disease, and medical treatment), suggesting that accelerated aging in the different organ systems is linked. We explore this hypothesis of the multidimensionality of aging in a different paper^42^. Interestingly, accelerated abdominal aging is also correlated with facial aging.

In terms of environmental variables, we found that smoking and sedentary behavior (e.g time spent watching television, lack of strenuous physical activity) is associated with accelerated abdominal aging, in accordance with the unambiguous literature on the subject^43,44^. We found some diet variables to be associated with decelerated abdominal aging (e.g cereal intake, bread intake). More generally, having a stable weight was associated with decelerated aging. Alcohol had a mixed association, with champagne, white wine, beer, cider, and red wine intake being all associated with accelerated abdominal aging, while alcohol intake frequency was associated with decelerated abdominal aging, possibly reflecting the complex literature on the topic^45^. Socioeconomic status (e.g education, income) was also negatively correlated with accelerated abdominal aging. In a developed country such as the US, the richest 1% live more than a decade longer than their poorest 1% counterparts, on average (10.1 ± 0.2 years for females, 14.6 ± 0.2 years longer for males)^46^. This difference could be mediated by better access to healthcare and health literacy^47^.

We speculate on possible mechanisms of abdominal aging and future avenues of research. We identified two environmental factors linked to abdomen “aging”, including alcohol consumption and smoking behavior in our XWASs. Alcohol consumption and smoking are risk factors for chronic fibrosis of the liver and pancreas. As pointed out by a reviewer, liver, and pancreas stromal stellate cell response may be one path to liver and pancreatic aging. Stellate cells are Vitamin A/retinol storing cells^48^ that are in nascent numbers in a developed and “healthy” organ^48,49^, but proliferate when stimulated, putatively by environmental exposures such as smoking and alcohol^50^. These cells also may be a source of, or induce circulating cytokines proliferation, leading to possible liver and pancreas damage. The responses mediated by stellate cells have been connected to fibrosis and cancer both in the liver and pancreas^49,51^. It is unclear whether stromal stellate cells of the pancreas and liver can be detected, or are being detected, by the MRIs utilized in this study. Future lines of investigations should examine, perhaps with the use of emerging image segmentation approaches, the role of stellate cells and predicted liver and pancreas age. A second line of investigation includes whether accelerated abdomen age is associated with fibrosis and/or pancreatic or liver cancer.

Abdomen Age, for the most part, is correlated with some, but not all, age predictors measured on different organs and tissues. Specifically, we examined the predicted accelerated AbdAge versus predicted accelerated cardiac, brain, eye, and musculoskeletal aging age dimensions. The major conclusion of these findings is that AbdAge is coincident with cardiac-MRI-based aging to a moderate extent. That is, individuals who are predicted to have older AbdAge may have younger predicted MRI-based Cardiac Age, for example. However, this was an exception: Abdomen Age is independent of the predicted biological age of other organs (median correlation across all 28 aging phenotypes: 0.15). We examined genetic architecture shared between AbdAge and other accelerated aging phenotypes, hypothesizing that abdominal age may be genetically similar to other dimensions of aging, such as musculoskeletal aging. Specifically, abdominal and spinal accelerated age have a large and significant positive genetic correlation (0.57): the SNPs that are associated with AbdAge are also associated with spine aging. The genetic correlation was weaker for other musculoskeletal dimensions, such as knee and hip skeletal ages. We had limited data to support the shared genetic architecture between AbdAge and other MRI-based aging phenotypes. AbdAge and cardiac phenotypic aging highlights potential co-incident aging; however, the genetic correlation findings between AbdAge and musculoskeletal age phenotypes indicate shared biology with spinal aging, but not hip or knee, may explain the phenotypic correlation. Another explanation may be that the deep learning algorithm may be identifying physiological features in common in the AbdAge and musculoskeletal images. To note, other indicators of aging biology, such as telomere length, exhibit substantial variation across tissues^52^, and Demanelis and colleagues observed a near zero correlation between pancreas and skeletal tissue telomere length.

We found that accelerated abdominal aging is heritable (h_g_^2^ = 26.3 ± 1.9%) and identified GWA-significant signals across all three phenotypes in non-coding or intergenic regions. For example, we found loci associated with AbdAge and Pancreas Age in genes or in genomic locations associated with another phenotype of aging, age-related macular degeneration (e.g.^53^). GWA in this region has been complex to untangle^54^ One of the genes, *PLEKHA1*, has exhibited pleiotropy and also been connected with type 2 diabetes^55^ and weight and height^56^ in new massive and multi-ethnic studies of the disease. Of interest, Sakaue et al^56^ accounted for non-linear interactions with age and sex and age-squared in estimating the associations. Second, we found an intergenic locus of the gene *EFEMP1* in both AbdAge and Liver Accelerated Age phenotypes. Other loci in this gene have been associated with other dimensions of aging, including “premature” aging and white matter density (a risk marker for dementia)^57,58^. Further, like *PLEKHA1*, *EFEMP1* is also connected to adiposity and body fat distribution^59^.

In conclusion, our biological age predictor can be used to assess abdominal aging and defines an accelerated aging phenotype that may be linked to disease and complications. The GWAS signals may also hint at possible new therapeutic gene targets for intervention or new instruments to study causality. Regarding the latter, one approach we aim to embark on is “Mendelian Randomization”^60,61^ where genetic variants for one trait (e.g., AbdAge) are associated with the genetic variants of another trait (e.g., cancer or type 2 diabetes) to causally infer the connection between them. Additionally, our predictor could be used on clinical trials to assess the effect of emerging rejuvenating therapies^62^ on abdominal organs and tissue. Other age predictors such as the DNA methylation clock are already leveraged to this end^18,63,64^ but, as aging is multidimensional^42,65^, diverse predictors will be needed to fully measure the therapeutic effect of candidate drugs on the different organs and tissues.

## Methods

We confirm that our research complies with all ethical regulations and is approved by UK Biobank (project ID: 52887) and was deemed not human subjects research by Harvard IRB (IRB16-2145) as defined by DHHS or FDA regulations; subjects are deidentified by the UK Biobank and we, the investigators, had no contact with the subjects.

### Cohort dataset: participants of the UK Biobank

We leveraged the UK Biobank^25^ cohort (project ID: 52887). The UKB cohort consists of data originating from a large biobank collected from 502,211 de-identified participants in the United Kingdom that were aged between 37 years and 74 years at enrollment (starting in 2006). Out of these participants, 44,481 had liver MRIs collected from them, and 36,591 had pancreas MRIs collected from them. The Harvard internal review board (IRB) deemed the research as non-human subjects research (IRB: IRB16-2145).

### Data types and preprocessing

#### Demographic variables

First, we removed out the UKB samples for which age or sex was missing. For sex, we used the genetic sex when available, and the self-reported sex when genetic sex was not available. We computed age as the difference between the date when the participant attended the assessment center and the year and month of birth of the participant to estimate the participant’s age with greater precision. We one-hot encoded ethnicity.

#### Liver and pancreas MRIs

UKB contains Liver MRI images (field 20204, 45,685 samples for 43,267 participants) of dimensions 288*384, stored as DICOM files. We removed the 83 images for which the image quality indicator had any flag on (field 22414). We applied an adaptive histogram equalizer filter to the images to enhance the contrast. We kept both images, which we named “Raw” and “Contrast”. We cropped off the legend on the right side of the images which yielded images of dimensions 288*350, that we stored as.jpg images. The UKB also contains pancreas images (field 20259, 37,619 samples for 35,285 participants). We followed the same pipeline used for the preprocessing of the liver images to preprocess the pancreas images and obtained 36,784 images. A sample of preprocessed abdominal (liver and pancreas) images can be found in Fig. 1A.

#### Data augmentation

To prevent overfitting and increase our sample size during the training we used data augmentation^66^ on the images. Each image was randomly shifted vertically (maximal amplitude ±10%) and horizontally (maximal amplitude ±10%), as well as rotated (maximal angle ±10 degrees). We chose the hyperparameters for these transformations’ distributions to represent the variations we observed between the images in the initial dataset. For example, we observed similar variation between images in the vertical and the horizontal direction, so both the random vertical and horizontal shifts were sampled from the [−10%, +10%] uniform distribution.

The data augmentation process is dynamically performed during the training. Augmented images are not generated in advance. Instead, each image is randomly augmented before being fed to the neural network for each epoch during the training.

### Machine learning algorithms

#### Convolutional neural networks architectures

We used transfer learning^67–69^ to leverage two different convolutional neural networks^70^ [CNN] architectures pre-trained on the ImageNet dataset^71–73^ and made available through the python Keras library^74^: InceptionV3^75^ and InceptionResNetV2^76^. We considered other architectures such as VGG16^77^, VGG19^77^, and EfficientNetB7^78^, but found that they performed poorly and inconsistently on our datasets during our preliminary analysis and we therefore did not train them in the final pipeline. For each architecture, we removed the top layers initially used to predict the 1000 different ImageNet images categories. We refer to this truncated model as the “base CNN architecture”.

We added to the base CNN architecture what we refer to as a “side neural network”. A side neural network is a single fully connected layer of 16 nodes, taking the sex and the ethnicity variables of the participant as input. The output of this small side neural network was concatenated to the output of the base CNN architecture described above. This architecture allowed the model to consider the features extracted by the base CNN architecture in the context of the sex and ethnicity variables. For example, the presence of the same anatomical feature can be interpreted by the algorithm differently for a male and for a female. We added several sequential fully connected dense layers after the concatenation of the outputs of the CNN architecture and the side neural architecture. The number and size of these layers were set as hyperparameters. We used ReLU^79^ as the activation function for the dense layers we added, and we regularized them with a combination of weight decay^80,81^ and dropout^82^, both of which were also set as hyperparameters. Finally, we added a dense layer with a single node and linear activation to predict age.

#### Compiler

The compiler uses gradient descent^83,84^ to train the model. We treated the gradient descent optimizer, the initial learning rate, and the batch size as hyperparameters. We used mean squared error [MSE] as the loss function, root mean squared error [RMSE], as the metric and we clipped the norm of the gradient so that it could not be higher than 1.0^85^.

We defined an epoch to be 32,768 images. If the training loss did not decrease for seven consecutive epochs, the learning rate was divided by two. This is theoretically redundant with the features of optimizers such as Adam, but we found that enforcing this manual decrease of the learning rate was sometimes beneficial. During training, after each image has been seen once by the model, the order of the images is shuffled. At the end of each epoch, if the validation performance improved, the model’s weights were saved.

We defined convergence as the absence of improvement on the validation loss for 15 consecutive epochs. This strategy is called early stopping^86^ and is a form of regularization. We requested the GPUs on the supercomputer for ten hours. If a model did not converge within this time and improved its performance at least once during the ten hours period, another GPU was later requested to reiterate the training, starting from the model’s last best weights.

### Training, tuning and predictions

We split the entire dataset into ten data folds by randomly assigning each participant into a fold. We manually tuned some of the hyperparameters before performing a simple cross-validation. We describe the tuning procedures in greater detail in the Supplementary Methods.

### Interpretability of the machine learning predictions

To interpret the models, we used attention maps (saliency and Grad-RAM). See Supplementary Methods.

### Ensembling to improve prediction and define aging dimensions

We built a three-level hierarchy of ensemble models to improve prediction accuracies. At the lowest level, we combined the predictions from different algorithms on the same dataset. For example, we combined the predictions generated by InceptionResNetv2 and Inceptionv3 from raw liver MRI images into a single raw liver MRI-based prediction. At the second level, we combined the predictions from the different preprocessing (raw and contrasted images) into a prediction for a specific organ (liver or pancreas). For the third and highest level, we combined all predictions into a general abdomen-based prediction. The ensemble models from the lower levels are hierarchically used as components of the ensemble models of the higher models. For example, the ensemble model built by combining the algorithms trained on raw liver MRIs is leveraged when building the general abdominal aging ensemble model.

We built each ensemble model separately on each of the ten data folds. For example, to build the ensemble model on the testing predictions of the data fold #1, we trained and tuned an elastic net on the validation predictions from the data fold #0 using a 10-folds inner cross-validation, as the validation predictions on fold #0 and the testing predictions on fold #1 are generated by the same model. We used the same hyperparameters space and Bayesian hyperparameters optimization method as we did for the inner cross-validation we performed during the tuning of the non-ensemble models.

To summarize, the testing ensemble predictions are computed by concatenating the testing predictions generated by ten different elastic nets, each of which was trained and tuned using a 10-folds inner cross-validation on one validation data fold (10% of the full dataset) and tested on one testing fold. This is different from the inner-cross validation performed when training the non-ensemble models, which was performed on the “training+validation” data folds, so on 9 data folds (90% of the dataset).

### Evaluating the performance of models

We evaluated the performance of the models using three different metrics: R-Squared [R^2^], root mean squared error [RMSE], and mean absolute error [MAE]. We computed a confidence interval on the performance metrics in two different ways. First, we computed the standard deviation between the different data folds. The test predictions on each of the ten data folds are generated by ten different models, so this measure of standard deviation captures both model variability and the variability in prediction accuracy between samples. Second, we computed the standard deviation by bootstrapping the computation of the performance metrics 1,000 times. This second measure of variation does not capture model variability but evaluates the variance in the prediction accuracy between samples.

### Abdominal age definition

We defined the abdominal age of participants for a specific abdominal dimension as the prediction outputted by the model trained on the corresponding dataset, after correcting for the bias in the residuals.

We indeed observed a bias in the residuals. For each model, participants on the older end of the chronological age distribution tend to be predicted younger than they are. Symmetrically, participants on the younger end of the chronological age distribution tend to be predicted older than they are. This bias does not seem to be biologically driven. Rather it seems to be statistically driven, as the same 60-year-old individual will tend to be predicted younger in a cohort with an age range of 60–80 years, and to be predicted older in a cohort with an age range of 40–60. We discuss the cause of this bias in the residuals more in detail in the supplementary. We ran a linear regression on the residuals as a function of age for each model and used it to correct each prediction for this statistical bias.

After defining biological age as the corrected prediction, we defined accelerated aging as the corrected residuals. For example, a 60-year-old whose liver MRI predicted an age of 70 years old after correction for the bias in the residuals is estimated to have a liver MRI-based abdominal age of 70 years, and an accelerated abdominal aging of ten years.

This step of correction of the predictions and the residuals takes place after the evaluation of the performance of the models but precedes the analysis of the abdominal ages properties.

### Correlation of abdomen, pancreas, and liver age with other accelerated age predictors

We correlated the abdomen age predicted outputs with the predicted output of 28 biological age predictors that we developed on other organ and organ systems, which include, Heart (MRI and ECG), Musculoskeletal (X-Ray), Arterial (Carotid ultrasound), Brain (MRI), eyes (OCT), and physiological measures (e.g., pulmonary function, blood laboratory values). For complex image data, we used a deep learning model building approach that is similar to that documented above^9,12,87,88^.

### Genome-wide association of accelerated abdominal aging

The UKB contains genome-wide genetic data for 488,251 of the 502,492 participants^89^ under the hg19/GRCh37 build. We used the average bias-corrected accelerated aging value (actual minus the predicted age) as the phenotype in the GWASs (see Supplementary Methods- Generating average predictions for each participant). Next, we performed genome-wide association studies [GWASs] to identify single-nucleotide polymorphisms [SNPs] associated with accelerated aging in each abdominal dimension using BOLT-LMM^90,91^ and estimated the the SNP-based heritability for each of our biological age phenotypes, and we computed the genetic pairwise correlations between dimensions using BOLT-REML^92,93^. We used the v3 imputed genetic data to increase the power of the GWAS, and we corrected all of them for the following covariates: age, sex, ethnicity, the assessment center that the participant attended when their DNA was collected, and the 20 genetic principal components precomputed by the UKB. We used the linkage disequilibrium [LD] scores from the 1000 Human Genomes Project^94^. To avoid population stratification, we performed our GWAS on individuals with White ethnicity.

#### Identification of SNPs associated with accelerated abdominal aging

We identified the SNPs associated with accelerated abdominal aging dimensions using the BOLT-LMM^90,91^ software (*p*-value of 5e-8). The sample size for the genotyping of the X chromosome is one thousand samples smaller than for the autosomal chromosomes. We, therefore, performed two GWASs for each aging dimension. (1) excluding the X chromosome, to leverage the full autosomal sample size when identifying the SNPs on the autosome. (2) including the X chromosome, to identify the SNPs on this sex chromosome. We then concatenated the results from the two GWASs to cover the entire genome, at the exception of the Y chromosome.

We used the Functional Mapping and Annotation (FUMA) software on the genome-wide association from each Abdomen-related aging phenotype (AbdAge, Pancreas and Liver Age)^95^ to identify (1) the loci associated with each of the traits, and the (2) nearest protein coding genes. We have also provided public links to the FUMA analyses, located here: AbdAge: https://fuma.ctglab.nl/browse/400, Liver Age: https://fuma.ctglab.nl/browse/401, and Pancreas Age: https://fuma.ctglab.nl/browse/402. We document our quality control procedure in the Supplementary Methods.

#### Heritability and genetic correlation

We estimated the heritability of the accelerated aging dimensions on the observed scale using the BOLT-REML^92^ software. We included the X chromosome in the analysis and corrected for the same covariates as we did for the GWAS. Using the same software and parameters, we computed the genetic correlations between accelerated aging in the two image-based abdominal dimensions and a priori accelerated aging phenotypes, including cardiac MRI^9^ and musculoskeletal (hip, spine, and knee) X-ray age predictors^12^.

We annotated the significant SNPs with their matching genes using the following four steps pipeline using the FUMA annotation software^95^.

### Non-genetic correlates of accelerated abdominal aging

We identified non-genetically measured (i.e factors not measured on a GWAS array) correlates of each aging dimension, which we classified in six categories: biomarkers, clinical phenotypes, diseases, family history, environmental, and socioeconomic variables. We refer to the union of these association analyses as an X-Wide Association Study [XWAS]. (1) We define as biomarkers the scalar variables measured on the participant, which we initially leveraged to predict age (e.g. blood pressure, Supplementary Data Table S2). (2) We define clinical phenotypes as other biological factors not directly measured on the participant but instead collected by the questionnaire, which we did not use to predict chronological age. For example, one of the clinical phenotypes categories is eyesight, which contains variables such as “wears glasses or contact lenses”, which is different from the direct refractive error measurements performed on the participants, which are considered “biomarkers” (Supplementary Data Table S5). (3) Diseases include the different medical diagnoses categories listed by UKB (Supplementary Data Table S8). (4) Family history variables include illnesses of family members (Supplementary Data Table S11). (5) Environmental variables include alcohol, diet, electronic devices, medication, sun exposure, early life factors, medication, sun exposure, sleep, smoking, and physical activity variables collected from the questionnaire (Supplementary Data Table S14). (6) Socioeconomic variables include education, employment, household, social support, and other sociodemographics (Supplementary Data Table S17). We provide information about the preprocessing of the XWAS in the Supplementary Methods.

### Reporting summary

Further information on research design is available in the Nature Research Reporting Summary linked to this article.

## Supplementary information

Supplementary Information

Reporting Summary

Description of Additional Supplementary Files

Supplementary Data Table S1

Supplementary Data Table S2

Supplementary Data Table S3

Supplementary Data Table S4

Supplementary Data Table S5

Supplementary Data Table S6

Supplementary Data Table S7

Supplementary Data Table S8

Supplementary Data Table S9

Supplementary Data Table S10

Supplementary Data Table S11

Supplementary Data Table S12

Supplementary Data Table S13

Supplementary Data Table S14

Supplementary Data Table S15

Supplementary Data Table S16

Supplementary Data Table S17

Supplementary Data Table S18

Supplementary Data Table S19

Supplementary Data Table S20

Supplementary Data Table 21

Supplementary Data Table S22

Supplementary Data Table S23

Supplementary Data Table S24

Supplementary Data Table S25

## Data Availability

The data are available by request from UK Biobank but are not available freely due to data privacy laws; for access, see https://www.ukbiobank.ac.uk/enable-your-research/apply-for-access. The processed age predictions will be available at request from UK Biobank and will be browsable in the catalog. The results can be interactively and extensively explored at https://www.multidimensionality-of-aging.net/, a website where we display and compare the performance and properties of the different biological age predictors we built. Select “Abdomen” as the aging dimension on the different pages to display the subset of the results relevant to this publication. The GWAS results (and summary statistics via FigShare) can be found here: AbdAge: https://fuma.ctglab.nl/browse/400 (via FigShare: 10.6084/m9.figshare.19361999 and https://figshare.com/articles/dataset/GWAS_Age_Abdomen_X_bgen_stats_gz/19361999), Liver Age: https://fuma.ctglab.nl/browse/401 (via FigShare: 10.6084/m9.figshare.19361972 and https://figshare.com/articles/dataset/GWAS_Age_AbdomenLiver_X_bgen_stats_gz/19361972) and Pancreas Age: https://fuma.ctglab.nl/browse/402 (via FigShare: 10.6084/m9.figshare.19361957 and https://figshare.com/articles/dataset/GWAS_Age_AbdomenPancreas_X_bgen_stats_gz/19361957).

## Code availability

Our code can be found on github: https://github.com/Deep-Learning-and-Aging. For the genetics analysis, we used the BOLT-LMM^90,91^ (v. 2.3.2) and BOLT-REML^92^ (v. 2.3.2), and FUMA (v1.3.7) software. The software versions for the deep learning pipeline are listed here and in the Supplementary Information: https://github.com/alanlegoallec/Multidimensionality_of_Aging/blob/main/Core_and_Images_pipeline/requirements.txt.
