# Supplementary material for: Using deep learning to predict abdominal age from liver and pancreas magnetic resonance images
Source: Nat Commun. 2022 Apr 13;13:1979. doi: 10.1038/s41467-022-29525-9 (PMC9007982; doi:10.1038/s41467-022-29525-9)
Supplement: Supplementary file 1 — Supplementary Information [file 41467_2022_29525_MOESM1_ESM.pdf]

Supplementary Methods, Figures, Tables and References

Using deep learning to predict abdominal age from liver and pancreas magnetic resonance images

Alan Le Goallec[1,2], Samuel Diai[1], Sasha Collin[1], Jean-Baptiste Prost[1], Théo Vincent[1], Chirag J. Patel[1*]

[1]Department of Biomedical Informatics, Harvard Medical School, Boston, MA, 02115, USA
[2]Department of Systems, Synthetic and Quantitative Biology, Harvard University, Cambridge, MA, 02118, USA
*Corresponding author

Contact information:
Chirag J Patel
chirag_patel@hms.harvard.edu

Table of contents

Abstract **1**

Introduction **2**

Results **5**

We predicted age within less than four years 5

Identification of features driving abdominal age prediction 6

Genetic factors and heritability of accelerated abdominal aging 8

Biomarkers, clinical phenotypes, diseases, environmental and socioeconomic variables associated with accelerated abdominal aging 12

Biomarkers associated with accelerated abdominal aging 13

Clinical phenotypes associated with accelerated abdominal aging 14

Diseases associated with accelerated abdominal aging 15

Environmental variables associated with accelerated abdominal aging 15

Socioeconomic variables associated with accelerated abdominal aging 16

Predicting accelerated abdominal aging from biomarkers, clinical phenotypes, diseases, environmental variables and socioeconomic variables 17

Phenotypic, genetic, and environmental correlation between liver MRI-based and pancreas MRI-based accelerated abdominal aging 18

Discussion **20**

Methods **26**
Data and materials availability 26
Software 26
Cohort Dataset: Participants of the UK Biobank 27
Data types and Preprocessing 27
Demographic variables 27
Liver and Pancreas MRIs 27
Data augmentation 28
Machine learning algorithms 28
Convolutional Neural Networks Architectures 28
Compiler 29
Training, tuning and predictions 30
Interpretability of the machine learning predictions 31
Ensembling to improve prediction and define aging dimensions 31
Evaluating the performance of models 32
Abdominal age definition 33
Correlation of Abdomen, Pancreas, and Liver Age with other accelerated age predictors 34
Genome-wide association of accelerated abdominal aging 34
Identification of SNPs associated with accelerated abdominal aging 35
Heritability and genetic correlation 36
Non-genetic correlates of accelerated abdominal aging 36

Author Contributions **38**

Acknowledgments **39**

Conflicts of Interest **39**

Funding **39**

References **40**

Table of contents 1

Results 3
Biological age does not outperform chronological age as a survival predictor 4
Quality control and power of genome-wide association studies 4

Discussion 5
Analysis of the bias in the residuals 5
Biological age does not outperform chronological age as a survival predictor 7

Methods 8
Hardware 8
Software 8

Training, tuning and predictions 9
Hyperparameters tuning upstream of the cross-validation 9
Cross-validation 12
Cross-validation example 13
Generating average predictions for each participant 14
Interpretability of the predictions 15
Genome-wide association study (GWAS) of accelerated aging 18
Non-genetic correlates of accelerated aging 19
Imputation of the non-genetic X-variables 19
X-Wide Association Studies 21
Prediction of accelerated abdominal aging 21
Algorithms 22
Training and tuning of the models 22
Nested cross-validation 22
Bayesian hyperparameters optimization 23
Example 24
Interpretability of the models 26
Phenotypic and Genetic Correlation 27
X-Correlations between the abdominal aging dimensions 27
X-Correlations based on the XWAS results 27
X-Correlations based on the feature importances 27
Survival prediction 28

Supplementary Figures 29

Supplementary Tables 36

Supplementary References 36

Results

Biological age does not outperform chronological age as a survival predictor

We predicted survival from biological age (Concordance Index=60.7+-24.5%) and found that it did not perform significantly differently from chronological age as a survival predictor (Concordance Index difference=0.6+-15.6%);, however, this result is based on a small number of deceased participants (n=21).

Quality control and power of genome-wide association studies

We observed that lambda GC increased as a function of minor allele frequency and INFO scores (Supplementary Figure 4). This trend is to be expected for a complex trait. We also observed that the lambdaGC are non-substantial at lower allele frequencies and INFO scores (Supplementary Figure 4). The lambda value (lambdaGC) for the GWASs were acceptable and were 1.04, 1.04, and 1.03 for the Abdomen (AbdAge), Liver, and Pancreas accelerated age phenotypes respectively.

We estimated power by using the GCTA-GREML power calculator from Visscher and colleagues [1]. For an approximate sample size of 30,000 and heritability of 0.3, we estimate greater than 80% power (99%) for detection of common SNPs at genome-wide level of significance ($p < 5e-8$). We set that the variance of the off-diagonal components of the genetic relationship matrix to be 2e-5.

Discussion

Analysis of the bias in the residuals

We observed a bias in the residuals for all models. Participants on the younger end of the cohort's chronological age distribution tend to be predicted older than they are, whereas participants on the older end of the distribution tend to be predicted younger than they are. This can be observed in other publications, as well[2–4]. It could be tempting to interpret this bias as a sign that young people tend to be accelerated agers and that old people tend to be decelerated agers. We however concluded that this observation is solely a statistical artifact after making the following four observations. (1) The bias is relative. If a model is trained on participants aged 20 to 50, the participants aged 50 years will tend to be predicted younger than they are. If we train a second model on participants aged 50 to 80, the same participants aged 50 years will tend to be predicted older than they are. No age group is intrinsically accelerated aging or decelerated aging, the bias for an age group depends on the position of the age group relative to the cohort on which the model was trained. This can for example be observed in the models trained on different age groups by Sagers et al.[3]. (2) A similar pattern is observed for all aging dimensions. (3) The bias gets smaller as the model's accuracy increases. (4) We predicted other quantitative variables such as serum cholesterol and blood pressure, and we found a similar bias pattern. Aycheh et al. also interpreted the bias as a statistical artefact and hypothesized that it was the result of a sample size imbalance between the different age groups[5]. We refuted this hypothesis by training models on datasets with different chronological age distributions (e.g. uniform, Gaussian), as the bias was largely unaffected by these preprocessing steps. We therefore formulate a hypothesis that is coherent with the five aforementioned observations. The default behavior of a model to minimize the loss function (mean squared error) if it is not provided with informative predictors with respect

to the target is to predict the mean of the target variable for every sample. Therefore, every participant that is younger than the mean cohort age will be predicted to be older than they are, and inversely for the older participants. The bias will also be proportional to the difference between the age of the participant and the mean age, which is what we observe in our models. As the model starts extracting relevant knowledge from the predictors, it must balance the potential improvement for the new prediction with the safety of its initial default guess, which behaves like a Bayesian prior, "pulling" every sample's prediction towards the mean target value on the dataset. Therefore, the bias remains approximately proportional to the difference between the participant's chronological age and the cohort's mean age, while progressively decreasing in amplitude as the R^2 increases.

Some other researchers who built biological age predictors by training models to predict chronological age reported this bias[5–7], but, to the best of our knowledge, most did not, although it can be observed in the scatter plot when one is present in the publication[2–4,8–17]. In some publications the bias does not seem to be present[18–22], one explanation being that, as the R^2 value of the model increases, it becomes harder to discern it. The bias is may also influence associations of the biological age predictor with mortality and diseases when not corrected and we recommend that researchers report it when they observe it in the future.

One key point we would like to highlight here is that it is crucial to correct for the bias in the residuals when defining accelerated aging before performing the association with survival, diseases, biomarkers or other phenotypes. If that is not done, older participants' biological age will tend to be lower than their chronological age, making them decelerated agers. In contrast, younger participants will tend to be accelerated agers. Because older participants are at a higher risk for death than younger participants, not accounting for the bias will likely lead to spurious and counterintuitive findings, such as decelerated aging being associated with increased mortality.

Biological age does not outperform chronological age as a survival predictor

Biological age did not perform significantly differently from chronological age as a survival predictor (Concordance Index difference=-0.6+-15.6%), which can in part be explained by the small number of UKB participants deceased at the time of our study. Specifically, out of the 34,445 participants who had MRIs collected from both their liver and their pancreas, only 21 were deceased. We emphasize, however, that one key argument is aging is not only about higher risk for death, but accruing age-related perturbations as one grows older that predisposes one to death. Concretely, for example, as a human liver or pancreas accrues exposures and diseases between 20 and 45 years in a person's life is likely not connected to the risk for death at those ages, which is already quite low. For example, in meta-analyses across 23 studies and ~42K participants, the association between epigenetic methylation-based biological age and mortality has been deemed “inconclusive” [23].

Methods

Hardware

We performed the computation for this project on Harvard Medical School's compute cluster, with access to both central processing units [CPUs] and general processing units [GPUs] (Tesla-M40, Tesla-K80, Tesla-V100) via a Simple Linux Utility for Resource Management [SLURM] scheduler.

Software Versions

We coded the deep learning in Python [24] (version 3.6) and used the following libraries: NumPy [25,26], Pandas [27], Matplotlib [28], Plotly [29], Python Imaging Library [30], SciPy [31–33], Scikit-learn [34], LightGBM [35], XGBoost [36], Hyperopt [37], TensorFlow 2 [38], Keras [39], Keras-vis [40], iNNvestigate [41]. We used Dash [42] to code the website on which we shared the results. We set the seed for the os library, the numpy library, the random library and the tensorflow library to zero. The software versions for the deep learning pipeline are listed here: https://github.com/alanlegoallec/Multidimensionality_of_Aging/blob/main/Core_and_Images_pipeline/requirements.txt and here:

```
Python version 3.6.
beautifulsoup4==4.8.2
bioinfokit==0.8.8
bs4==0.0.1
efficientnet==1.1.0
gpuinfo==1.0.0a6
GPUtil==1.4.0
graphviz==0.13.2
hyperopt==0.1.2
imageio==2.5.0
innvestigate==1.0.8
ipdb==0.13.2
keract==4.0.0
Keras==2.3.1
Keras-Applications==1.0.8
Keras-Preprocessing==1.1.0
keras-vis==0.4.1
kerasplotlib==0.1.4
lifelines==0.25.6
```

lightgbm==2.3.1
matplotlib==3.3.3
matplotlib-venn==0.11.5
more-itertools==7.2.0
multiprocess==0.70.11.1
numpy==1.18.5
nvgpu==0.7.0
nvidia-ml-py==375.53.1
opencv-python==4.1.1.26
opt-einsum==3.2.1
pandas==0.25.3
pickleshare==0.7.5
Pillow==8.0.1
pydicom==1.3.0
scikit-image==0.14.2
scikit-learn==0.23.0
scipy==1.4.1
seaborn==0.9.0
six==1.12.0
sklearn==0.0
tensorflow-addons==0.10.0
tensorflow-estimator==2.2.0
tensorflow-gpu==2.2.0
tf-estimator-nightly==1.14.0.dev2019030115
tf-keras-vis==0.3.1
threadpoolctl==2.0.0
utils==1.0.1
virtualenv==15.1.0
xgboost==0.82

Our code can be found on github: https://github.com/Deep-Learning-and-Aging. The versions of the libraries are in the Supplement and in the repository. For the genetics analysis, we used the BOLT-LMM (v. 2.3.2) and BOLT-REML (v. 2.3.2) and FUMA (v1.3.7) software. We coded the parallel submission of the jobs in Bash.

Training, tuning and predictions

Hyperparameters tuning upstream of the cross-validation

The hyperparameters we tuned were the number of added fully connected dense layers, the number of nodes in these layers, their activation function, the optimizer, the initial learning rate, the weight decay, the dropout rate, the data augmentation amplitude and the batch size. Repeatedly tuning the values of the hyperparameters for different deep neural networks architectures and on the different cross-validation folds would have been prohibitively time and

resource consuming. Instead, we sequentially explored how each hyperparameter was affecting the training and validation performances for a single architecture (InceptionV3) on a single cross validation fold (fold #0, see Methods - Training, tuning and predictions - Images - Cross-validation for the detailed description of the cross-validation). We then extrapolated the hyperparameter values to the other architectures, datasets and cross-validation folds. The hyperparameters combinations tested during the tuning can be found in Supplementary Data Table S22.

First, we maximized the batch size for each architecture. The maximum number of images per batch depends on the memory of the GPU and the size of the architecture, which itself depends on the dimensions of the image. We used a batch size of 32 for InceptionV3 and 8 for InceptionResNetV2.

Then, we tested the learning rates, including 1e-6, 1e-5, 1e-4, 1e-3, 1e-2 and 1e-1. We observed that learning rates larger than 1e-4 prevented the model from converging for some runs. Second, we did not observe significant differences between the results obtained with learning rates smaller than 1e-4. We therefore set the initial learning rate to be 1e-4 for all models to shorten the time to convergence while ensuring that the learning rate was small enough to allow convergence and the finding of a local minima for the loss function.

Then we tested three different optimizers to perform the gradient descent: Adam [43], Adadelta [44] and RMSprop [45]. We did not observe any significant differences between the optimizers, so we set the optimizer to be Adam.

We then added different numbers of fully connected layers between the base CNN and side CNN’s concatenated outputs and the final activation layer. We set the number of nodes to be 1,024 in the first added layer and then decreased the number of nodes by a factor of two for each

successive layer. For example, if we added three fully connected layers, the number of nodes was 1024, 512 and 256. We added zero, one and five layers. We did not observe significant differences in the performance of the different architectures, so we set the number of fully connected layers to one.

We then tested powers of two from 16 to 2,048 as the number of nodes in this single layer. We did not observe significant differences between these architectures, so we set the number of nodes to be 1,024 to keep the number close to the initial number of nodes in the imported CNN architectures, as these were initially used to perform classification between 1,000 categories.

We tested two different activation functions for the activation functions of the fully connected layers we added in the side neural network and before the final linear layer. We did not observe any significant differences between the rectified linear units [ReLU] [46] and the scaled exponential linear units [SELU] [47] as activation functions, so we used the more common ReLU.

We then tested different levels of data augmentation. We introduced a hyperparameter that we called “data augmentation factor”. The data augmentation factor modulates the amount of variation introduced by the data augmentation, while preserving the ratio between the different transformations. For example, a data augmentation factor of one is equivalent to the default data augmentation (see Preprocessing - Data augmentation - Images), but a data augmentation factor of two will double the ranges of the possible values sampled and the expected values for the vertical shift, the horizontal shift, the rotation and the zoom on the original images. We tested the following values for the data augmentation factor: 0, 0.1, 0.5, 1, 1.5 and 2. We found that different values for the data augmentation factor hyperparameter yielded similar results, as long as the data augmentation factor was not zero. We therefore set the data augmentation factor to be one when training the final models.

We then tuned the dropout rate for the fully connected layers we added. We tested the following values: 0, 0.1, 0.25, 0.3, 0.5, 0.75, 0.9 and 0.95. We observed that a dropout rate of 0.95 led to underfitting and that smaller values reduced overfitting on the training set but without improving the validation performance. As a consequence, we used a dropout rate of 0.5.

Finally, we tuned the weight decay. We tested the following values: 0, 0.1, 0.2, 0.3, 0.4, 0.5, 1, 5, 10 and 100. For the larger datasets, we found that weight decay values as low as 0.4 could lead to underfitting. We found that lower weight decay values reduced overfitting on the training set without significantly improving the validation performance. We set the weight decay to 0.1. Altogether, we found that hyperparameter tuning had little effect on the validation performance as long as extreme hyperparameters values were not selected.

Cross-validation

Training deep convolutional neural networks on images and videos is too time and resource consuming to perform a nested cross-validation. Therefore, we tuned the hyperparameters during the preliminary analysis, as described above. After hyperparameters tuning, we performed a simple outer cross-validation to obtain a testing prediction for each sample of the datasets, but we replaced the inner cross-validation with a simple split between the training fold and the validation fold (Supplementary Data Table S23). Although the hyperparameters were already tuned, a validation set was still required for two reasons: (1) to perform early stopping [48], a form of regularization. (2) to generate a set of validation predictions that are necessary for efficient ensemble building (see Methods - Models ensembling) and model selection. During the cross-validation, we scaled and centered the target variable (chronological age) as well as the side predictors (sex and ethnicity) around zero with a standard deviation of one, using the training

summary statistics. Scaling the target and the input helps prevent the issues of exploding and vanishing gradients [49,50].

Cross-validation example

For the sake of clarity, let us walk through an example. Let us say that we want to generate unbiased predictions for every sample in a dataset using a CNN. First, we select the data fold #0 as the validation set, the data fold #1 as the testing set, and the remaining data folds (#2-9) as the training set. Then we scale and center the target (age), and the side predictors (sex and ethnicity) using the training mean and standard deviation: for each of the variables, we subtract the training mean to the variable on both the training, the validation and the testing set, and we divide it by the training standard deviation. We then train the model on the training set until convergence and select the architecture's parameters (also known as "weights") associated with the epoch that yielded the lowest validation RMSE. We then use the optimal weights to generate validation predictions for the data fold #0 and testing predictions on the data fold #1. Finally, we unscale the validation and testing predictions by multiplying them by the initial age training standard deviation before adding the initial age training mean to them. This completes the first cross-validation fold.

We then reiterate the process, this time using the data fold #1 as the validation set, the data fold #2 as the testing set, and the remaining data folds (#0 and #3-9) as the training set. We use the optimized weights to generate the validation predictions on the data fold #2, and the testing predictions on the data fold #3. We unscale the validation and testing predictions. This completes the second cross-validation fold. We reiterate the process eight more times to complete the cross-validation. We then concatenate the validation predictions from the ten data folds to obtain the final validation predictions, and the testing predictions from the ten data folds to obtain the final testing predictions.

Generating average predictions for each participant

We generated an average prediction for each individual, reported to UKB's instance 0. We walk through an example. Let us assume a participant had liver MRI samples collected from them in instances 2 and 3, respectively at age 70 and 80. Let us assume that the age predictions were respectively 64 and 78, so the residuals are respectively -6 years and -2 years, for an average of -4 years. However, we still need to take into account the bias in the residuals, defined as the difference between the participant's chronological age and the prediction. As explained in more details under Methods - Biological age definition, we observed a bias in the residuals as a function of chronological age. Participants on the younger end of the chronological age distribution tend to be predicted older than they actually are, whereas participants on the older end of the distribution tend to be predicted younger than they actually are. We need to properly account for this bias when translating a prediction from a more recent instance to an older instance. Let us assume that the average bias in the residuals for participants who are 70 and 80 years old is respectively -2 years and -4 years. After correcting for this bias, the predictions are now respectively 64-(-2)=66 and 78-(-4) = 82. Therefore, the corrected residuals for this participant are respectively -4 years and +2 years, for an average of -1 years. Finally, let us assume that the participant was 60 years old in instance 0. We will assign a single prediction of 60-1=59 years to the participant, but we still need to un-correct for the bias in residuals. Let us assume that the average bias for the residuals at age 60 is +5 years. We will assign a final prediction for the participant of 59+5=64 years. This new set of predictions reported on the instance 0 is more likely to have a non-zero sample size overlap with other predictors based on datasets collected on instance 0 (e.g. blood biochemistry) and can therefore be leveraged by the XWAS analysis.

A key point we would like to highlight here is that we did not actually correct for the bias in the residuals at this step of the pipeline. Instead, we corrected then un-corrected the predictions that

we translated from different instances to the instance 0. The actual correction for the residual biases takes place when defining the biological age phenotypes (see Methods - Biological age definition).

To distinguish between raw predictions on the instance 0, and the average predictions reported to the instance 0, we created a new instance which we named instance “*”. We refer to these predictions as “participants predictions”, as opposed to “samples predictions”.

Interpretability of the predictions

To interpret the CNNs built on images, we first used saliency maps [51], which we coded using the keract python library. For each input sample, a saliency map uses the gradient of the final prediction with respect to each individual input pixel to estimate whether changing the value of this pixel would affect the prediction. Pixels for which the gradient is close to zero are not important, whereas pixels with a large gradient are estimated to be important.

We then built a second attention map using a custom version of the Gradient-weighted Class Activation Mapping [Grad-CAM] algorithm [52] adapted to regression rather than multi-class classification: Gradient-weighted Regression Activation Mapping [Grad-RAM]. The intuition behind Grad-CAM maps is that they are similar to saliency maps [52], but instead of computing the gradient with respect to the input image, they compute it with respect to the activation of the last convolutional layer. As convolutional layers maintain the spatial organization of the input image, Grad-CAM can still identify which region of the image is driving the predictions. Because Grad-CAM does not have to backpropagate the gradient all the way back to the input image, it is considered a less noisy alternative to the saliency maps. In the same way that saliency maps need to combine the attention maps generated in the different input channels (e.g. RGB) into a

single activation map, Grad-CAM must combine the attention maps generated on the different filters of the last convolutional layer. For example, the last convolutional layer for InceptionResNetV2 has 1,792 filters. Grad-CAM combines these 1,792 attention maps into a single attention map using a linear combination. In the initial Class Activation Mapping [CAM] algorithm [53], generating CAM activation maps required to retrain the model after modifying the architecture and replacing all the fully connected layers after the final convolutional layer with a global max pooling operation, which converted each filter into a scalar feature. The intuition behind this substitution was that each filter could be interpreted as detecting a specific feature, and global max pooling yielded a scalar that could be interpreted as the presence (high value) or absence (low value) of the feature anywhere on the image. The scalar values were then linearly combined and activated using the softmax function to yield the probabilities of belonging to different classes. To obtain the activation map for a specific class, the filters of the last convolution layer were linearly combined using the weights connecting the scalar features obtained after the max pooling operation to the final prediction score for that class. CAM was later improved to become Grad-CAM [52]. Grad-CAM saves the need for modifying the architecture of the model and retraining it by approximating the linear regression weight for each final convolutional filter by the mean activation gradient over the pixels of the filter. The intuition behind this approximation is that a filter's pixel is important if changing its value affects the final prediction, so a high average gradient over the pixels of the filter justifies that this filter should be given a higher weight when merging all the filters into a single attention map. To adapt Grad-CAM to our regression task we (1) computed the derivatives of the chronological age prediction rather than a class' prediction and (2) removed the ReLU activation applied to the weighted sum of the last convolutional filters, which we replaced by an absolute value. The rationale is that for (Grad-)CAM maps, we only want to highlight the regions of the picture which are associated with a high probability for the class. In contrast, for (Grad-)RAM we care as much about the regions of the input image that can strongly increase the chronological age prediction as about the regions that can strongly decrease it.

Because the filters in the last convolutional layer are the result of the processing of the input image by several convolutional layers with possibly negative weights, the sign of the last convolutional layer's pixels and regression weights cannot be linked to either accelerated aging or decelerated aging, only to the magnitude of the shift that would affect the prediction if each region of the input image was modified. Regression Activation Mapping (RAM) was mentioned as a possible extension of CAM in the original CAM publication [53] and has been used to interpret models CNNs built on retinal images [54] and cortical surfaces [55], but we are to our knowledge the first to describe the generalization of Grad-CAM to a regression task. One notable difference between our implementation and Wang and Yang's implementation [54] is that we are taking the absolute value of the final attention map, as mentioned above. We found that not taking the absolute value led to misleading attention maps for participants with high chronological age predictions. The attention map highlights important areas with negative values, which are therefore depicted in blue, a color otherwise associated with unimportant regions in traditional CAMs. Inversely, regions on the input image for which the attention map has a slight positive value are spuriously considered to be the most important and are highlighted in red. We therefore advise that RAM or Grad-RAM be implemented using an absolute value. We coded Grad-RAM using the get_activations and get_gradients_of_activations functions of the keract python library.

It is important to understand that unlike the feature importances described under "Scalar data-based predictors", which describe the model itself, attention maps are sample specific. In other words, they can be used to explain which features drove the predictions for a specific inputted sample but cannot provide an explanation for the way the model is performing predictions in general.

For each aging subdimension, we generated the attention maps for the best performing CNN architecture. We selected representative samples for which we computed the different attention

maps. We computed attention maps for the two sexes (female and male), for three age ranges (ten youngest ages, ten middle ages and ten oldest ages of the chronological age distribution) and for three aging rates (accelerated agers, normal agers, decelerated agers). For each intersection of the three categories listed above, we selected the ten most representative samples (e.g. the ten most accelerated agers among young males). The figures in this paper only present the first, most representative of these ten samples. The complete set of samples can be found on the website.

Genome-wide association study (GWAS) of accelerated aging

As in the main Methods, we used the bias-corrected accelerated age (chronological minus predicted age) as the phenotype in the GWASs. We used the Functional Mapping and Annotation (FUMA, v 1.3.7) software on the genome wide association from each Abdomen-related aging phenotype (AbdAge, Pancreas and Liver Age) [56] to identify (1) the loci associated with each of the traits, and the (2) nearest protein coding genes. We have also provided public links to the FUMA analyses, located here: AbdAge: https://fuma.ctglab.nl/browse/400, Liver Age: https://fuma.ctglab.nl/browse/401, and Pancreas Age: https://fuma.ctglab.nl/browse/402. Briefly, to identify the significant locus, SNPs are filtered that have a GWAS-level of significance (in our study, $p < 5e-8$). SNPs that are GWA-significant and have a r^2 greater than 0.6 are candidate SNPs for a locus; other SNPs are considered independent. The SNP with the lowest p-value and independent of other SNPs at a r^2 less than 0.1 is the “lead SNP”. We report the lead SNP and the number of other SNPs in linkage with the lead SNP. Next, to identify closest protein coding genes, FUMA uses ANNOVAR[57], to positionally map SNPs.

We conducted a few steps for GWAS quality control (QC), guided by the steps taken by Dr. Ben Neale (http://www.nealelab.is/blog/2017/9/11/details-and-considerations-of-the-uk-biobank-

gwas). First, we restricted the GWAS to have INFO scores greater than 0.3 and minor allele frequency of 0.001. The INFO score measures the certainty of the imputation and ranges from 0 (zero certainty) to 1. Imputation was performed by UK Biobank. Second, we estimated the lambda GC in different bins of the INFO and minor allele frequency spectrum.

Non-genetic correlates of accelerated aging

Unlike DNA, biomarkers, phenotypes, diseases, family history, environmental variables and socioeconomics can change over life. As a consequence, we compared each biomarker, phenotype and environmental variable with the accelerated aging of the participant at the time the exposure was measured and we used the “Samples predictions”, as opposed to the “Participants predictions” that we used for the identification of genetic correlates (see Methods - Models ensembling - Generating average predictions for each participant).

Imputation of the non-genetic X-variables

Most X-variables were not collected on all four instances. Additionally, no X-variables were collected at the same time as the accelerometer data was collected. To identify the non-genetic correlates of accelerated aging, we had to impute the values of the X-variables for the ages of the participants for which they were not available. We considered two imputation methods, which we refer to as the “cross-sectional” and the “longitudinal” imputations.

For the cross-sectional imputation, we computed a linear regression for each X variable as a function of age, adjusting for sex. We then used the slope of the linear regression to extrapolate the value of the XWAS variable at different ages.

For the longitudinal imputation, we first selected, for each X variable, all the participants that had at least two measures taken for this X variable. We then performed a linear regression for each participant. We then averaged the slope of the linear regressions over all the participants of the same sex. Finally, we used this slope to extrapolate the value of the XWAS variable at different ages for all participants depending on their sex, in the same way we did it for the cross-sectional imputation.

It is important to notice that for both the cross-sectional imputation and the longitudinal imputation, data can only be imputed when the XWAS variable has been measured at least once for the participant. This raw measure is then used to extrapolate which value the X variable was likely taking a couple years earlier and/or later.

The advantage of the cross-sectional imputation is larger sample sizes. The advantage of the longitudinal method is that it corrects for generational effects. For example, old people have shorter legs than young people on average [58]. This is not because human legs shrink as we grow older. Instead, people who are old today already had shorter legs when they were young. If the cross-sectional regression is used to impute the length of the participants on instances where it was not measured, it will spuriously assign smaller values to the older samples. In contrast, the longitudinal regression learns the regression coefficient by comparing each participant to themselves as they age and will therefore not capture the generational effect. When used to predict the participants legs' length, it will impute constant values over time. To evaluate which of the two imputation methods should be preferred, we used them to predict X-variables for which we knew the actual values and computed the R-Squared values associated with the predictions. We found that, even with sample sizes as small as 200 samples, longitudinal imputation outperformed cross-sectional imputation. We therefore used longitudinal imputation.

X-Wide Association Studies

First, we tested for associations in an univariate context by computing the partial correlation between each X-variable and abdominal aging dimensions. To compute the partial correlation between an X-variable and an aging, we followed a three steps process. (1) We ran a linear regression on each of the two variables, using age, sex and ethnicity as predictors. (2) We computed the residuals for the two variables. (3) We computed the correlation between the two residuals and the associated p-value if their intersection had a sample size of at least ten samples. We used a threshold for significance of 0.05 and corrected the p-values for multiple testing using the Bonferroni correction. We plotted the results using a volcano plot. We refer to this pipeline as an X-Wide Association study [XWAS].

In the supplementary tables and the results, we rank the X-variables subcategories by decreasing percentage of variables associated with accelerated aging (note that the ranking is therefore biased towards categories with fewer variables). For each subcategory, we list the three most associated variables, based on the absolute value of the correlation coefficient. For the exhaustive list, please refer to https://www.multidimensionality-of-aging.net/xwas/univariate_associations.

Prediction of accelerated abdominal aging

We predicted accelerated aging for the different abdominal aging dimensions as a function of the biomarkers, clinical phenotypes, diseases, family history, environmental and socioeconomic variables. We leveraged the same pipeline to identify which features were driving the predictions. We built a model for each X-variables subcategory (Supplementary Data Table S2, Supplementary Data Table S5, Supplementary Data Table S8, Supplementary Data Table S11, Supplementary Data Table S14, Supplementary Data Table S17).

Algorithms

We used three different algorithms. Elastic Nets [EN] (a regularized linear regression that represents a compromise between ridge regularization and LASSO regularization), Gradient Boosted Machines [GBM] (LightGBM implementation [35]), and Neural Networks [NN]. The choice of these three algorithms represents a compromise between interpretability and performance. Linear regressions and their regularized forms (LASSO [59], ridge [60], elastic net [61]) are highly interpretable using the regression coefficients but are poorly suited to leverage non-linear relationships or interactions between the features and therefore tend to underperform compared to the other algorithms. In contrast, neural networks [62,63] are complex models, which are designed to capture non-linear relationships and interactions between the variables. However, tools to interpret them are limited [64] so they are closer to a "black box". Tree-based methods such as random forests [65], gradient boosted machines [66] or XGBoost [36] represent a compromise between linear regressions and neural networks in terms of interpretability. They tend to perform similarly to neural networks when limited data is available, and the feature importances can still be used to identify which predictors played an important role in generating the predictions. However, unlike linear regression, feature importances are always non-negative values, so one cannot interpret whether a predictor is associated with older or younger age. We also performed preliminary analyses with other tree-based algorithms, such as random forests [65], vanilla gradient boosted machines [66] and XGBoost [36]. We found that they performed similarly to LightGBM, so we only used this last algorithm as a representative for tree-based algorithms in our final calculations.

Training and tuning of the models

Nested cross-validation

Cross-validation is a method to tune the regularization of models and prevent overfitting [67]. For the models inputting scalar data (Figure 1A in green), we tuned the hyperparameters and

generated a testing prediction for each sample using a nested 10x9-folds cross-validation. We refer to the two nested cross-validations as the “outer” and the “inner” cross-validations. The outer-cross validation is used to generate an unbiased testing prediction for each sample, as opposed to a simple split of the data into a “training+validation” set on one hand, and a testing set on the other hand, which would only generate a testing prediction for one tenth of the dataset. The inner cross-validation is used to tune the hyperparameters more precisely, leveraging the full inner cross-validation dataset as a validation set, as opposed to a simple data split of the “training+validation” dataset into a training and a validation sets, which would only use one data fold as the validation set to estimate the performance associated with a specific combination of hyperparameters. The nested cross-validation is illustrated in Supplementary Data Table S25.

Bayesian hyperparameters optimization

To tune the hyperparameters, we used the Tree-structured Parzen Estimator Approach [68] [TPE] of the hyperopt python package [69]. TPE is a sequential Bayesian hyperparameters optimization method that iteratively suggests the next most promising hyperparameters combination as a function of the hyperparameters combinations that have already been tested, by building a probabilistic representation of the objective function. We set the number of iterations to 30. For each model, 30 different hyperparameter combinations are iteratively tested before selecting the best performing one. The hyperparameters names and their ranges defining the hyperparameters space can be found in Supplementary Data Table S24. It might be of interest to other researchers that we initially tuned the hyperparameters using a random search [70] with the same number of iterations, and we did not observe a significant improvement in the model’s performance after implementing the Bayesian hyperparameters optimization.

Example

For the sake of clarity, let us walk through a concrete example, which is illustrated in Supplementary Data Table S25. Suppose we want to generate unbiased predictions for every sample in a dataset using an elastic net. First, let us generate the testing prediction for the data fold F9, which is performed by the first fold of the outer cross-validation (outer cross-validation fold 0). We select the data fold F9 out of the ten data folds as the testing fold, and we select the remaining nine data folds as "training+validation" folds for the inner cross-validation. We scale and center the target (age) and the predictors using the mean and standard deviation values of the variables on the "training+validation" dataset. We then enter the first inner-cross validation.

For the first inner cross-validation fold, we select the data fold F8 as the validation set, and the remaining eight "training+validation" data folds as the training set. We re-scale and center age and the predictors in the training and the validation sets using the mean and standard deviation values of the training set. We train the model on the eight training data folds with the first hyperparameters combination sampled by the TPE algorithm (one value for alpha and one value for l1_ratio) and generate validation predictions on the validation fold (data fold F8), which we unscale. This completes the first of the nine-inner cross-validation folds (Inner CV fold 0). We then permute the nine inner data folds. We scale the age and the predictors using the mean and standard deviation computed on the new training set. Then we train the model with the same first combination of hyperparameters on eight data folds, leaving aside the data fold F9 (still being used as the testing set for the outer cross-validation) and the data fold F7 (now being used as the validation set for the inner cross-validation). We then use the new trained model to generate validation predictions on the data fold F7, which we unscale. This completes the second of the nine inner-cross validation folds (Inner CV fold 1). We then reiterate these inner permutation and training processes seven more times, until every data fold in the nine "training+validation" data

folds is used as the validation set once. At this point, we concatenate the validation predictions from these nine validation folds to obtain the overall validation predictions associated with the first hyperparameters combination, and compute the associated performance metric (e.g. RMSE). This completes the inner-cross validation for the first hyperparameters combination.

We then perform the same 9-folds inner cross-validation, this time with the second hyperparameters combination suggested by the TPE algorithm. We iterate this process 28 more times, until 30 different hyperparameters combinations have iteratively been tested. Next, we select the hyperparameter combination that yielded the best validation performance (e.g. minimum RMSE), and we retrain a model on the whole nine "training+validation" data folds (all data folds except for data fold #1), using this best performing hyperparameters combination. This completes the first inner cross-validation.

We then use the model to generate unbiased predictions on the unseen testing set (data fold F9) and record these predictions. By anticipation for the ensembling algorithm (see Methods - Models ensembling) we also need to compute validation predictions on the data fold F8. We do this by training a model on all the data folds aside from the validation fold (data fold F8) and the testing fold (data fold F9), with the selected hyperparameters combination. We then use this trained model to compute predictions on the validation fold (data fold F8) and record these predictions, after unscaling them. This completes the first of the ten outer cross-validation folds (outer cross-validation 0).

We then complete the second outer cross-validation fold (outer cross-validation 1), this time using the data fold F8 as the testing dataset, to obtain unbiased testing predictions on this data fold, as well as validation predictions on the data fold F7. We reiterate the process eight more times to obtain the testing and validation predictions on the remaining data folds. We then concatenate the testing predictions from the ten data folds to obtain our final testing predictions for the model.

Similarly, we concatenate the validation predictions from the ten data folds to obtain our final testing predictions for the model, which will later be used during ensemble models building and model selection (see Methods - Models ensembling).

The final validation and testing predictions for each data fold are therefore not necessarily associated with the same hyperparameters combination. It is also important to notice that we performed a single outer cross-validation, but that we performed a separate inner-cross validation for each outer cross-validation fold (hence the word “nested”), for a total of ten inner cross-validations per outer cross-validation fold.

Interpretability of the models

For elastic nets, we interpreted the models using the values of the regression coefficients. Large absolute values for these coefficients means they played an important role when generating the predictions. For gradient boosted machines we used the feature importances, which are based on the number of times a tree selected each of the variables. Variables with high feature importances were selected more often and are therefore likely to play a key role in predicting chronological age. For neural networks, we estimated the importance of each feature by permuting it randomly between samples before computing the performance of the model. The score of each feature is the difference between the R-Squared value before and after the random permutations. Features whose random permutation leads to a large decrease in the model’s performance are estimated to be important predictors of chronological age.

We estimated the concordance between the three different algorithms by computing the Pearson and the Spearman correlations between their feature importances.

X-Correlations between the abdominal aging dimensions

X-Correlations based on the XWAS results

For the sake of clarity, let us walk through an example. We want to compute the environmental correlation between accelerated liver MRI-based and pancreas MRI-based aging. The XWAS generates a vector whose components are the partial correlations between the accelerated aging phenotype and each environmental variable, for both accelerated liver MRI-based and pancreas MRI-based aging. We compute three different Pearson correlations between these two partial correlation vectors. (1) The "All" correlation, using all the components of the two vectors; this correlation tends to be inflated by the large number of X-variables whose correlation with both accelerated aging dimensions is close to zero. (2) The "Intersection" correlation, using only the environmental variables that were significantly associated with both of the accelerated aging dimensions;because the cardinality of the intersection can be small, a small number of X-variables can yield very high or very low correlations. (3) The "Union" correlation, using only the environmental variables that were significantly associated with at least one of the two accelerated aging dimensions; the "Union" correlation represents a compromise between the "All" and the "Intersection" correlations. The figures in this paper were generated using the "Union" correlation, but all three correlations can be explored on the website.

X-Correlations based on the feature importances

We then computed the correlations between the feature importances for different accelerated aging dimensions to estimate the X-correlation between the different dimensions. We used the same method as described above under "X-Correlations based on the XWAS results", replacing the coefficient obtained for each X-variable in a univariate context (using partial correlation with accelerated aging) with the coefficient obtained in a multivariate context (as an accelerated aging predictor in a multivariate model).

Survival prediction

UKB collects mortality data from its 502,492 participants, 30,263 of which (6%) are already dead. We highlight the fact that these death events are surprisingly unevenly distributed between data modalities. For example, out of the 207,932 participants for whom pulse wave analysis data was recorded, only 6,000 are dead, half less than the 12,500 death events we would have observed if the death events were evenly distributed between the different datasets.

We leveraged the mortality data to compute the Concordance Index [CI] associated with each biological age definition. The CI measures whether the predictor successfully predicts which participants will die first by computing the percentage of participant pairs for which the participant with the higher biological age dies before the participant with a lower biological age. Accordingly, CI values are usually between 0.5 (useless survival predictor) and 1.0 (perfect survival predictor, at least in terms of ranking the death events). Chronological age is an effective survival predictor, so for each biological age dimension we computed the difference between the CI obtained using biological and the CI obtained using chronological age to estimate the added value contributed by the biomarkers used to define the biological age dimension.

We computed the standard deviations for the CI values using the same protocol as the one described under “Methods - Evaluating the performance of the models”. We computed an associated two-tailed p-value for each CI difference between the biological age dimension and chronological age assuming a Gaussian distribution and using the associated z-value.

Supplementary Figures

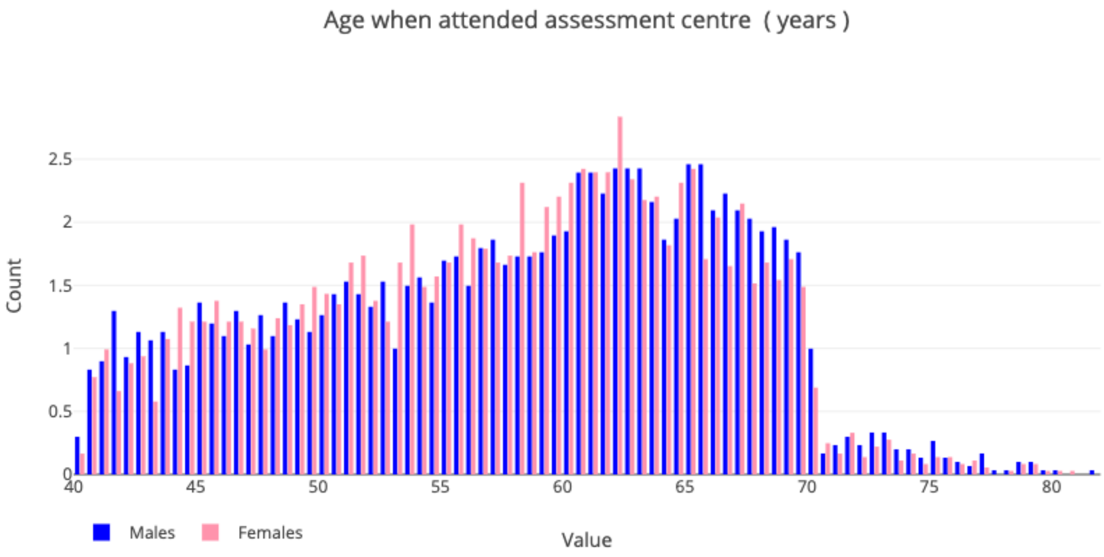

Supplementary Figure S1: Age Distribution of the UK Biobank cohort

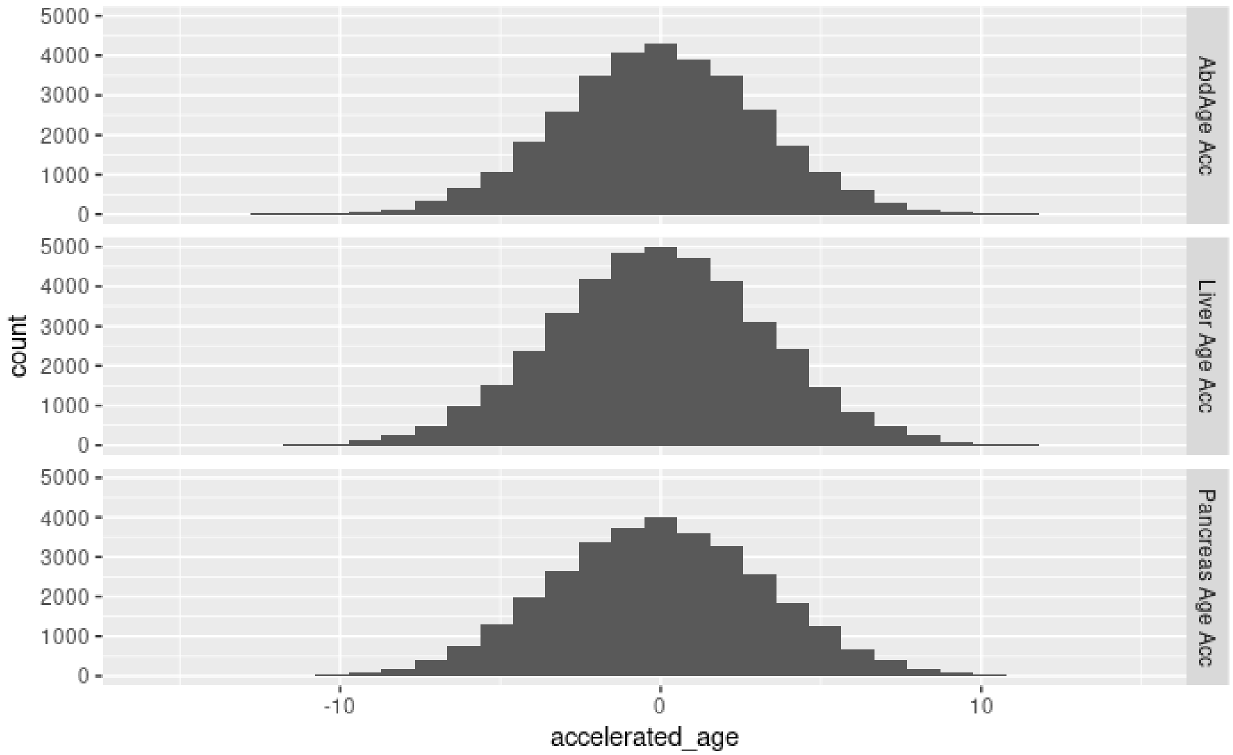

Supplementary Figure S2: Distribution of Accelerated Abdominal Age, Liver Age, and Pancreas Age.

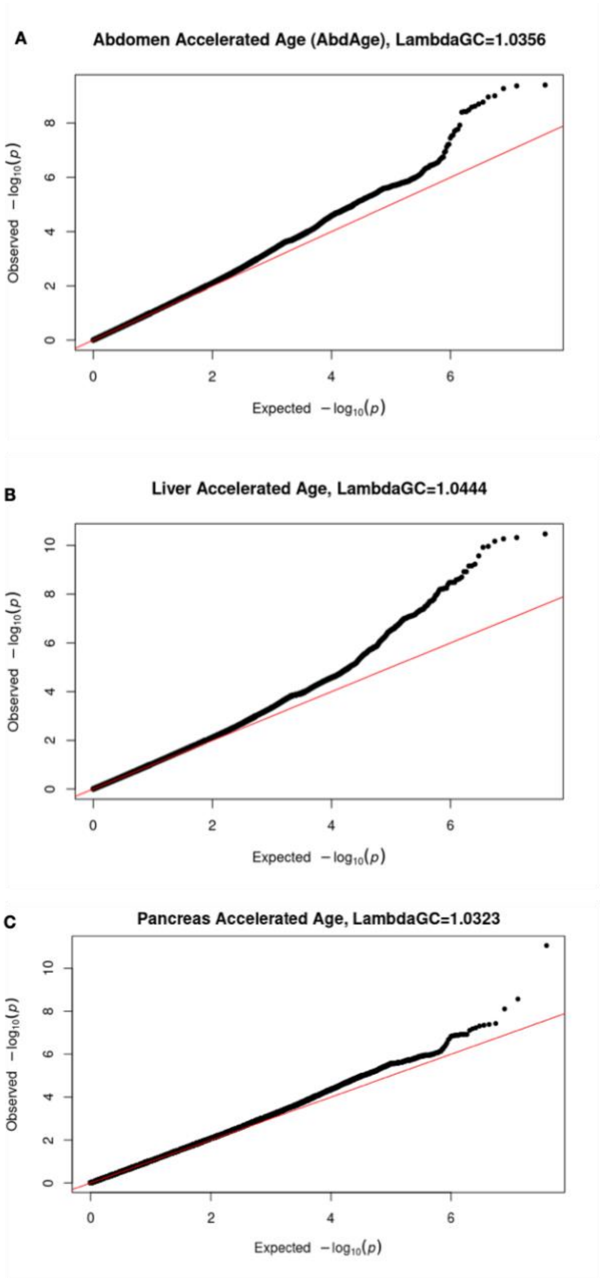

Supplementary Figure S3. GWAS quantile-quantile plots of association test statistics for A) AbdAge Acceleration, B) Liver Age Acceleration and C) Pancreas Age Acceleration. All p-values are two sided and not corrected for multiple comparisons.

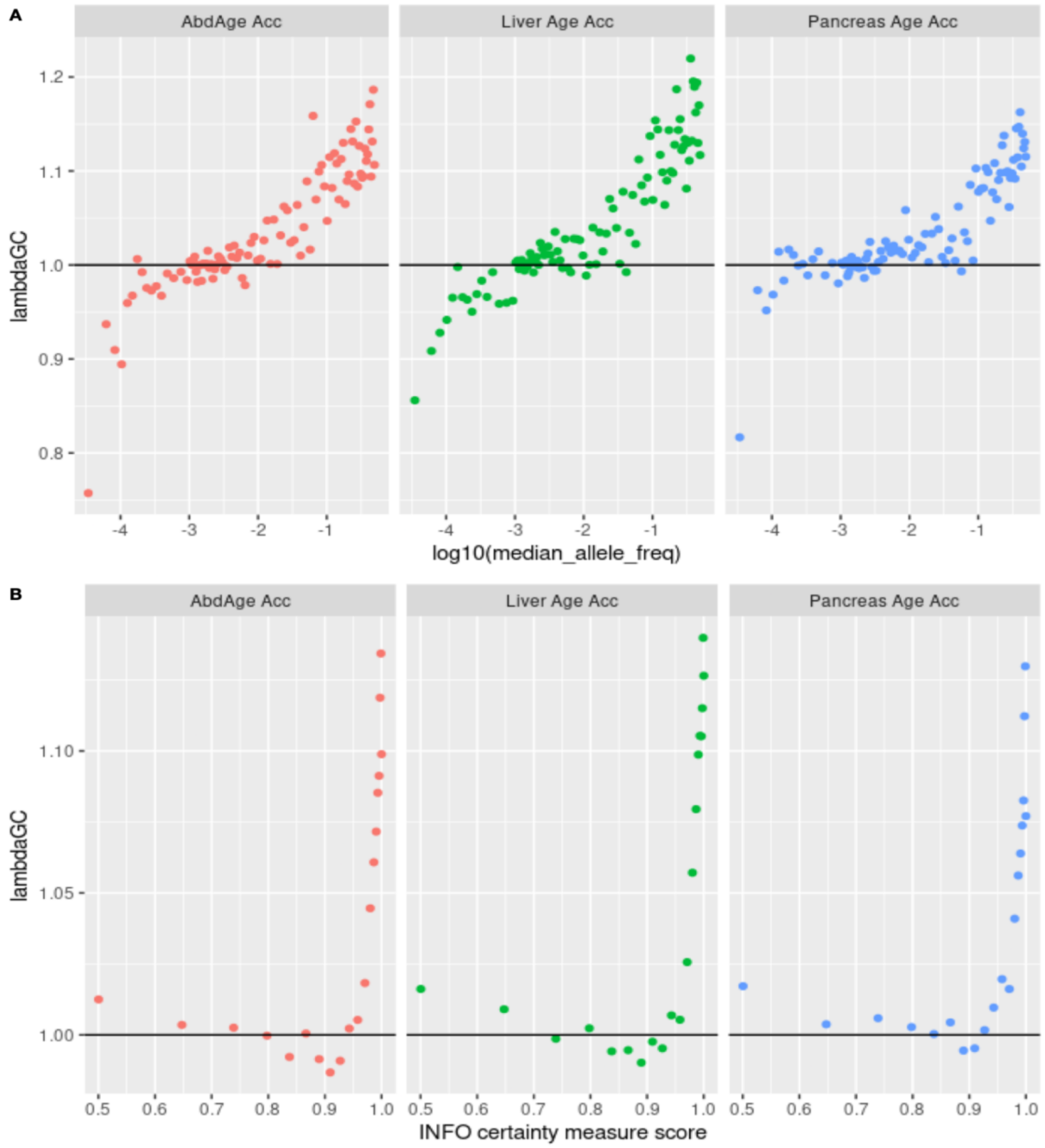

Supplementary Figure S4. A) log10(minor allele frequency) or B) INFO score vs lambda Genomic Control (lambdaGC).

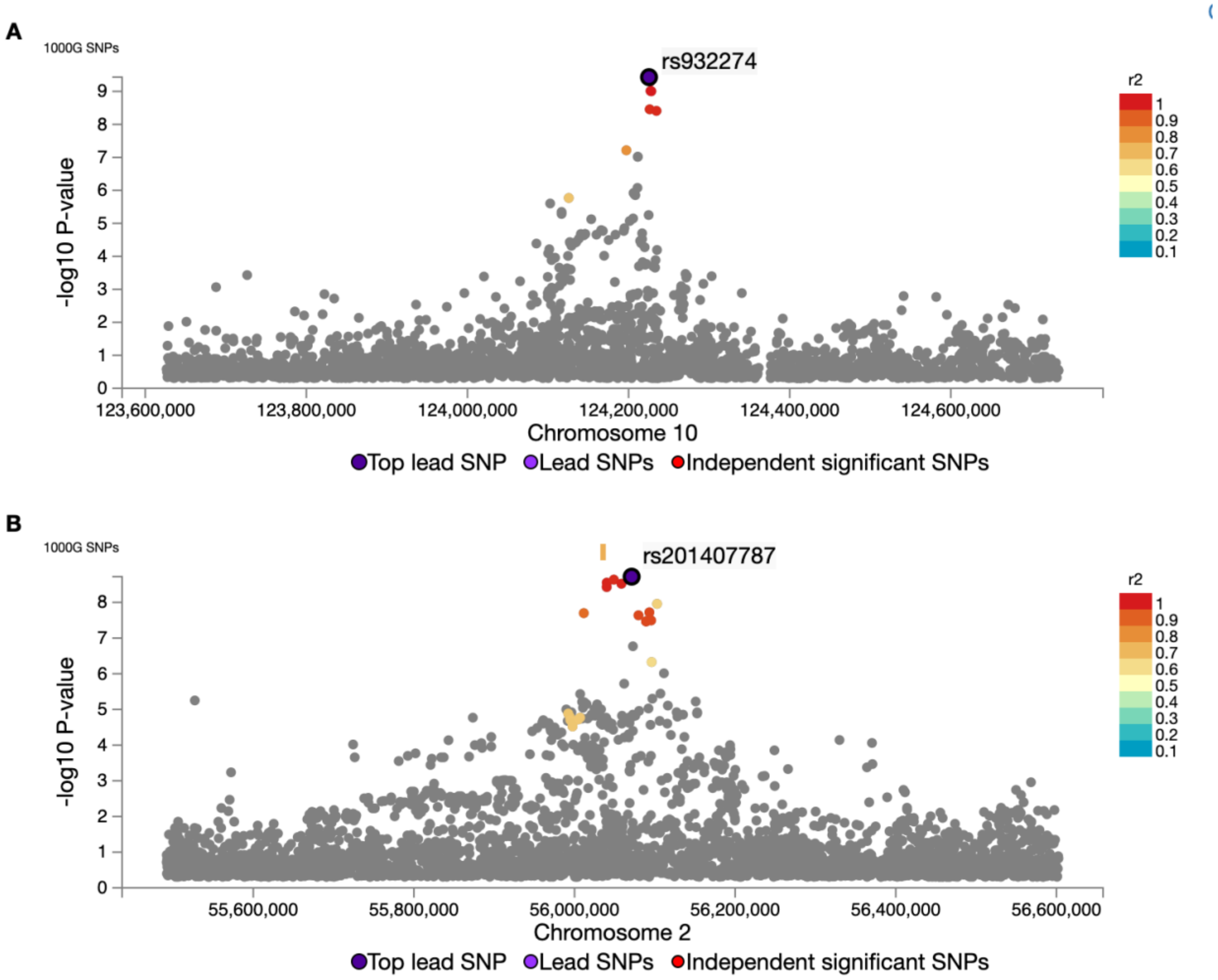

Supplementary Figure S5. Locuszoom plots for significant loci for AbdAge. r2 denotes SNP linkage disequilibrium (r2 of 1 denotes SNPs that are in full linkage). All p-values are two sided and not corrected for multiple comparisons.

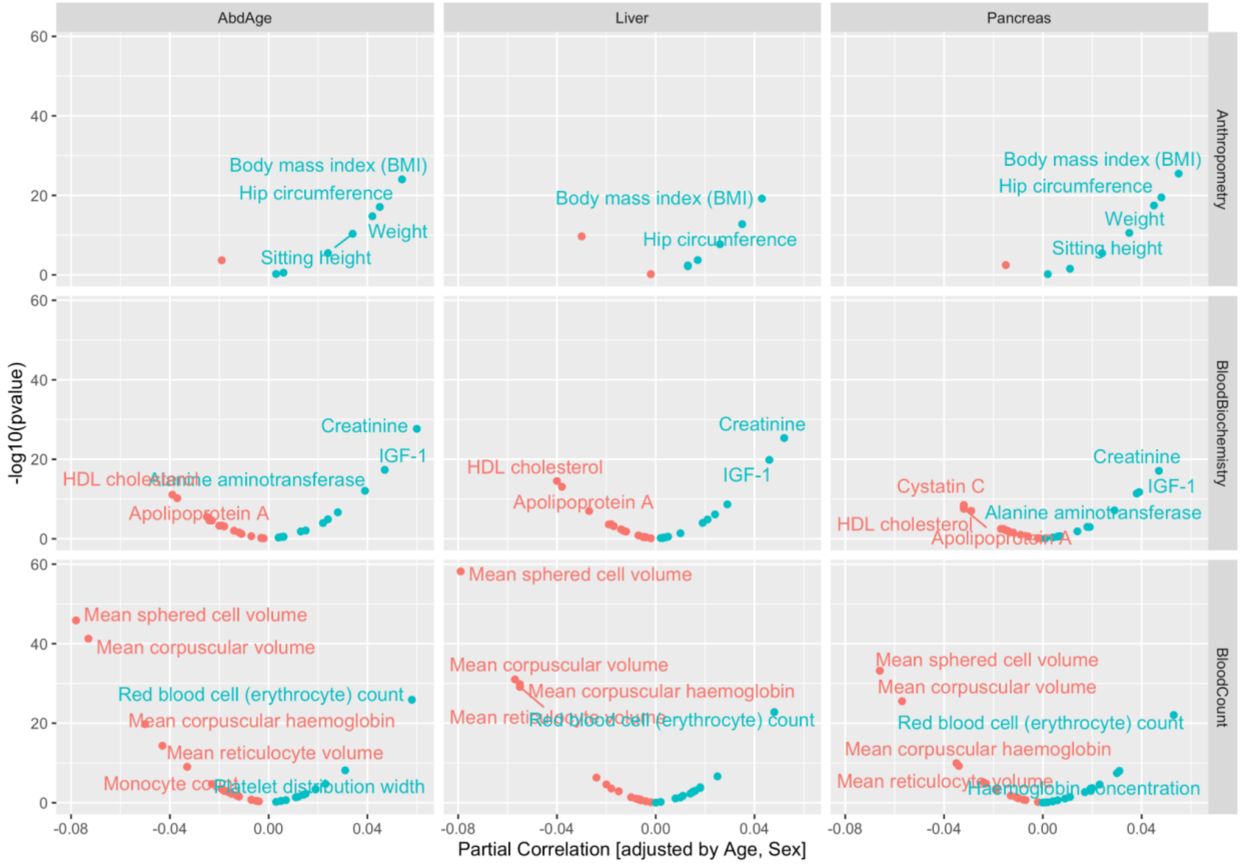

Supplementary Figure S6. Partial correlations between biomarkers of Anthropometry, Biochemistry, and Blood Count and General AbdAge, Liver Accelerated Age, and Pancreas Accelerated Age. Labeled partial correlations are shown (blue positive, red are negative) for those correlations that are greater than 0.03 and p value of correlation less than 1e-5. All p-values are two sided and not corrected for multiple comparisons.

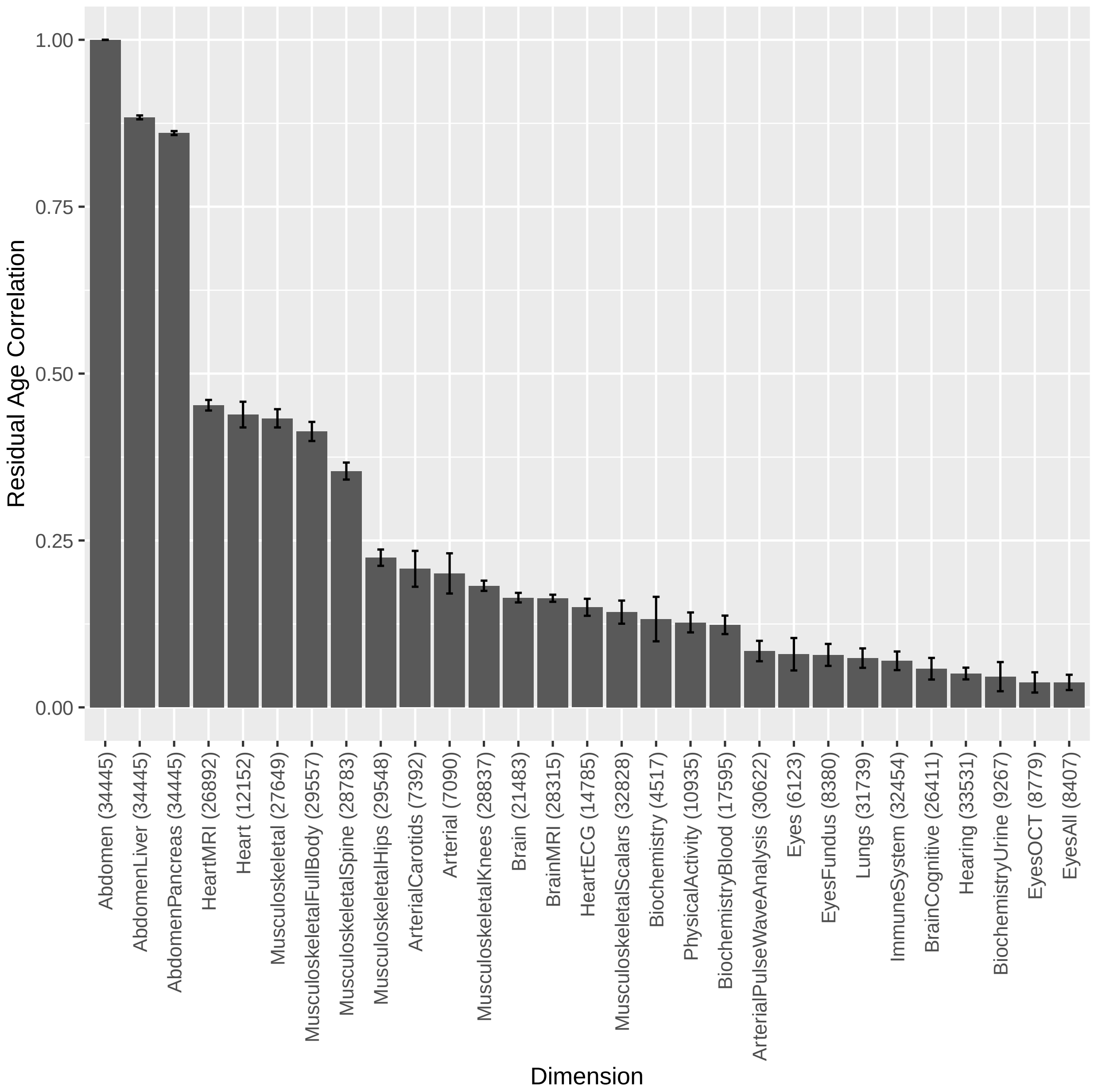

Supplementary Figure S7: Correlation between AbdAge Accelerated Age and other dimensions of accelerated age. Error bars are +/- 2 SD of the bootstrapped estimate. Sample sizes are in parenthesis.

Supplementary Tables

Supplementary Table S1**: GWASs summary - Heritability and number of GWA-significant loci associated with accelerated aging in each abdominal dimension**

Abdominal dimension	Sample size	Loci	Heritability (%± SE)	R-Squared for age prediction (%±SE)
Abdomen	32,475	3	26.3±1.9	76.3±0.2
Liver	40,760	11	22.3±1.5	71.5±0.2
Pancreas	32,548	2	22.1±1.9	70.3±0.3

Supplementary References

1. Visscher, P. M. *et al.* Statistical power to detect genetic (co)variance of complex traits using SNP data in unrelated samples. *PLoS Genet.* **10**, e1004269 (2014).
2. Attia, Z. I. *et al.* Age and Sex Estimation Using Artificial Intelligence From Standard 12-Lead ECGs. *Circ. Arrhythm. Electrophysiol.* **12**, e007284 (2019).
3. Sagers, L., Melas-Kyriazi, L., Patel, C. J. & Manrai, A. K. Prediction of chronological and biological age from laboratory data. *Aging* **12**, 7626–7638 (2020).
4. Putin, E. *et al.* Deep biomarkers of human aging: Application of deep neural networks to biomarker development. *Aging* **8**, 1021–1033 (2016).
5. Aycheh, H. M. *et al.* Biological Brain Age Prediction Using Cortical Thickness Data: A Large Scale Cohort Study. *Front. Aging Neurosci.* **10**, 252 (2018).
6. Langner, T., Wikstrom, J., Bjerner, T., Ahlstrom, H. & Kullberg, J. Identifying Morphological Indicators of Aging With Neural Networks on Large-Scale Whole-Body MRI. *IEEE Trans. Med. Imaging* **39**, 1430–1437 (2020).
7. Pardoe, H. R. & Kuzniecky, R. NAPR: a Cloud-Based Framework for Neuroanatomical Age Prediction. *Neuroinformatics* **16**, 43–49 (2018).
8. Liem, F. *et al.* Predicting brain-age from multimodal imaging data captures cognitive impairment. *Neuroimage* **148**, 179–188 (2017).
9. Zoubi, O. A. *et al.* Predicting Age From Brain EEG Signals—A Machine Learning Approach. *Frontiers in Aging Neuroscience* vol. 10 (2018).
10. Cole, J. H. *et al.* Predicting brain age with deep learning from raw imaging data results in a reliable and heritable biomarker. *Neuroimage* **163**, 115–124 (2017).
11. Varatharajah, Y. *et al.* Predicting Brain Age Using Structural Neuroimaging and Deep

Learning. doi:10.1101/497925.

12. Madan, C. & Kensinger, E. A. Predicting age from cortical structure across the lifespan. doi:10.1101/248518.
13. Poplin, R. *et al.* Predicting Cardiovascular Risk Factors from Retinal Fundus Photographs using Deep Learning.
14. Hoffmann, R., Lauterbach, C., Conradt, J. & Steinhage, A. Estimating a person's age from walking over a sensor floor. *Comput. Biol. Med.* **95**, 271–276 (2018).
15. Rahman, S. A. & Adjeroh, D. A. Deep Learning using Convolutional LSTM estimates Biological Age from Physical Activity. *Sci. Rep.* **9**, 11425 (2019).
16. Lehallier, B., Shokhirev, M. N., Wyss-Coray, T. & Johnson, A. A. Data mining of human plasma proteins generates a multitude of highly predictive aging clocks that reflect different aspects of aging. *Aging Cell* e13256 (2020).
17. Fleischer, J. G. *et al.* Predicting age from the transcriptome of human dermal fibroblasts. *Genome Biol.* **19**, 221 (2018).
18. Zhai, J. & Li, K. Predicting Brain Age Based on Spatial and Temporal Features of Human Brain Functional Networks. *Front. Hum. Neurosci.* **13**, 62 (2019).
19. Galkin, F. *et al.* Human Gut Microbiome Aging Clock Based on Taxonomic Profiling and Deep Learning. *iScience* **23**, 101199 (2020).
20. Pyrkov, T. V. *et al.* Extracting biological age from biomedical data via deep learning: too much of a good thing? *Sci. Rep.* **8**, 5210 (2018).
21. Enroth, S., Enroth, S. B., Johansson, Å. & Gyllensten, U. Protein profiling reveals consequences of lifestyle choices on predicted biological aging. *Sci. Rep.* **5**, 17282 (2015).
22. Mamoshina, P. *et al.* Machine Learning on Human Muscle Transcriptomic Data for Biomarker Discovery and Tissue-Specific Drug Target Identification. *Frontiers in Genetics* vol. 9 (2018).
23. Fransquet, P. D., Wrigglesworth, J., Woods, R. L., Ernst, M. E. & Ryan, J. The epigenetic

clock as a predictor of disease and mortality risk: a systematic review and meta-analysis. *Clin. Epigenetics* **11**, 62 (2019).

24. Van Rossum, G. & Drake, F. L. *The Python Language Reference Manual*. (Network Theory Limited, 2011).
25. Oliphant, T. E. *A guide to NumPy*. vol. 1 (Trelgol Publishing USA, 2006).
26. Walt, S. van der, van der Walt, S., Chris Colbert, S. & Varoquaux, G. The NumPy Array: A Structure for Efficient Numerical Computation. *Computing in Science & Engineering* vol. 13 22–30 (2011).
27. McKinney, W. & Others. Data structures for statistical computing in python. in *Proceedings of the 9th Python in Science Conference* vol. 445 51–56 (Austin, TX, 2010).
28. Hunter, J. D. Matplotlib: A 2D Graphics Environment. *Comput. Sci. Eng.* **9**, 90–95 (2007).
29. Inc, P. T. Collaborative data science. *Montreal: Plotly Technologies Inc Montral* (2015).
30. Clark, A. Pillow Python Imaging Library. *Pillow—Pillow (PIL Fork) 5. 4. 1 documentation* (2018).
31. Virtanen, P. *et al.* SciPy 1.0: fundamental algorithms for scientific computing in Python. *Nature Methods* vol. 17 261–272 (2020).
32. Oliphant, T. E. Python for Scientific Computing. *Computing in Science Engineering* **9**, 10–20 (2007).
33. Millman, K. J., Jarrod Millman, K. & Aivazis, M. Python for Scientists and Engineers. *Computing in Science & Engineering* vol. 13 9–12 (2011).
34. Pedregosa, F. *et al.* Scikit-learn: Machine learning in Python. *the Journal of machine Learning research* **12**, 2825–2830 (2011).
35. Ke, G. *et al.* LightGBM: A Highly Efficient Gradient Boosting Decision Tree. in *Advances in Neural Information Processing Systems 30* (eds. Guyon, I. et al.) 3146–3154 (Curran Associates, Inc., 2017).
36. Chen, T. & Guestrin, C. XGBoost: A Scalable Tree Boosting System. in *Proceedings of the*

22nd ACM SIGKDD International Conference on Knowledge Discovery and Data Mining 785–794 (Association for Computing Machinery, 2016).

37. Bergstra, J., Yamins, D. & Cox, D. D. Hyperopt: A python library for optimizing the hyperparameters of machine learning algorithms. in *Proceedings of the 12th Python in science conference* vol. 13 20 (Citeseer, 2013).
38. Abadi, M. *et al.* TensorFlow: Large-scale machine learning on heterogeneous systems. (2015).
39. Chollet, F. & Others. keras. (2015).
40. Kotikalapudi, R. & Others. keras-vis. 2017. *URL https://github. com/raghakot/keras-vis* (2019).
41. Alber, M. *et al.* iNNvestigate neural networks. *J. Mach. Learn. Res.* **20**, 1–8 (2019).
42. Hossain, S., Calloway, C., Lippa, D., Niederhut, D. & Shupe, D. Visualization of Bioinformatics Data with Dash Bio. in *Proceedings of the 18th Python in Science Conference* 126–133 (2019).
43. Kingma, D. P. & Ba, J. Adam: A Method for Stochastic Optimization. *arXiv [cs.LG]* (2014).
44. Zeiler, M. D. ADADELTA: An Adaptive Learning Rate Method. *arXiv [cs.LG]* (2012).
45. Hinton, G. Slide 29 of Lecture 6, Geoffrey Hinton coursera’s class. *http://www.cs.toronto.edu* http://www.cs.toronto.edu/~tijmen/csc321/slides/lecture_slides_lec6.pdf.
46. Nair, V. & Hinton, G. E. Rectified Linear Units Improve Restricted Boltzmann Machines. (2010).
47. Klambauer, G., Unterthiner, T., Mayr, A. & Hochreiter, S. Self-Normalizing Neural Networks. in *Advances in Neural Information Processing Systems 30* (eds. Guyon, I. et al.) 971–980 (Curran Associates, Inc., 2017).
48. Prechelt, L. Early Stopping - But When? in *Neural Networks: Tricks of the Trade* (eds. Orr, G. B. & Müller, K.-R.) 55–69 (Springer Berlin Heidelberg, 1998).

49. Hochreiter, S. Untersuchungen zu dynamischen neuronalen Netzen. *Diploma, Technische Universität München* **91**, (1991).

50. Hochreiter, S., Bengio, Y., Frasconi, P., Schmidhuber, J. & Others. Gradient flow in recurrent nets: the difficulty of learning long-term dependencies. (2001).

51. Alqaraawi, A., Schuessler, M., Weiß, P., Costanza, E. & Berthouze, N. Evaluating saliency map explanations for convolutional neural networks: a user study. in *Proceedings of the 25th International Conference on Intelligent User Interfaces* 275–285 (Association for Computing Machinery, 2020).

52. Selvaraju, R. R. *et al.* Grad-cam: Visual explanations from deep networks via gradient-based localization. in *Proceedings of the IEEE international conference on computer vision* 618–626 (2017).

53. Zhou, B., Khosla, A., Lapedriza, A., Oliva, A. & Torralba, A. Learning deep features for discriminative localization. in *Proceedings of the IEEE conference on computer vision and pattern recognition* 2921–2929 (2016).

54. Wang, Z. & Yang, J. Diabetic Retinopathy Detection via Deep Convolutional Networks for Discriminative Localization and Visual Explanation. *arXiv [cs.CV]* (2017).

55. Duffy, B. A. *et al.* Regression activation mapping on the cortical surface using graph convolutional networks. (2019).

56. Watanabe, K., Taskesen, E., van Bochoven, A. & Posthuma, D. Functional mapping and annotation of genetic associations with FUMA. *Nat. Commun.* **8**, 1826 (2017).

57. Wang, K., Li, M. & Hakonarson, H. ANNOVAR: functional annotation of genetic variants from high-throughput sequencing data. *Nucleic Acids Res.* **38**, e164 (2010).

58. Le Goallec, A. & Patel, C. J. Age-dependent co-dependency structure of biomarkers in the general population of the United States. *Aging* **11**, 1404–1426 (2019).

59. Tibshirani, R. Regression shrinkage and selection via the lasso. *J. R. Stat. Soc. Series B Stat. Methodol.* **58**, 267–288 (1996).

60. Hoerl, A. E. & Kennard, R. W. Ridge Regression: Biased Estimation for Nonorthogonal Problems. *null* **12**, 55–67 (1970).

61. Zou, H. & Hastie, T. Regularization and variable selection via the elastic net. *J. R. Stat. Soc. Series B Stat. Methodol.* **67**, 301–320 (2005).

62. Rosenblatt, F. *The Perceptron: A Theory of Statistical Separability in Cognitive Systems (Project Para)*. (Cornell Aeronautical Laboratory, 1958).

63. Popescu, M.-C., Balas, V. E., Perescu-Popescu, L. & Mastorakis, N. Multilayer perceptron and neural networks. *WSEAS Trans. Circuits and Syst.* **8**, (2009).

64. Ribeiro, M. T., Singh, S. & Guestrin, C. ' Why should I trust you?' Explaining the predictions of any classifier. in *Proceedings of the 22nd ACM SIGKDD international conference on knowledge discovery and data mining* 1135–1144 (2016).

65. Breiman, L. Random Forests. *Mach. Learn.* **45**, 5–32 (2001).

66. Friedman, J. H. Greedy Function Approximation: A Gradient Boosting Machine. *Ann. Stat.* **29**, 1189–1232 (2001).

67. Kohavi, R. & Others. A study of cross-validation and bootstrap for accuracy estimation and model selection. in *Ijcai* vol. 14 1137–1145 (Montreal, Canada, 1995).

68. Bergstra, J. S., Bardenet, R., Bengio, Y. & Kégl, B. Algorithms for Hyper-Parameter Optimization. in *Advances in Neural Information Processing Systems 24* (eds. Shawe-Taylor, J., Zemel, R. S., Bartlett, P. L., Pereira, F. & Weinberger, K. Q.) 2546–2554 (Curran Associates, Inc., 2011).

69. Bergstra, J., Yamins, D. & Cox, D. Making a Science of Model Search: Hyperparameter Optimization in Hundreds of Dimensions for Vision Architectures. in (eds. Dasgupta, S. & McAllester, D.) vol. 28 115–123 (PMLR, 2013).

70. Bergstra, J. & Bengio, Y. Random search for hyper-parameter optimization. *J. Mach. Learn. Res.* **13**, 281–305 (2012).
