# Supplementary material for: Using deep learning to predict abdominal age from liver and pancreas magnetic resonance images
Source: Nat Commun. 2022 Apr 13;13:1979. doi: 10.1038/s41467-022-29525-9 (PMC9007982; doi:10.1038/s41467-022-29525-9)
Supplement: Supplementary file 2 — Reporting Summary [file 41467_2022_29525_MOESM2_ESM.pdf]

nature portfolio

Corresponding author(s): Chirag J Patel

Last updated by author(s): Mar 15, 2022

Reporting Summary

Nature Portfolio wishes to improve the reproducibility of the work that we publish. This form provides structure for consistency and transparency in reporting. For further information on Nature Portfolio policies, see our Editorial Policies and the Editorial Policy Checklist.

Statistics

For all statistical analyses, confirm that the following items are present in the figure legend, table legend, main text, or Methods section.

n/a	Confirmed	
[ ]	[x]	The exact sample size (*n*) for each experimental group/condition, given as a discrete number and unit of measurement
[ ]	[x]	A statement on whether measurements were taken from distinct samples or whether the same sample was measured repeatedly
[ ]	[x]	The statistical test(s) used AND whether they are one- or two-sided<br>*Only common tests should be described solely by name; describe more complex techniques in the Methods section.*
[ ]	[x]	A description of all covariates tested
[ ]	[x]	A description of any assumptions or corrections, such as tests of normality and adjustment for multiple comparisons
[ ]	[x]	A full description of the statistical parameters including central tendency (e.g. means) or other basic estimates (e.g. regression coefficient) AND variation (e.g. standard deviation) or associated estimates of uncertainty (e.g. confidence intervals)
[ ]	[x]	For null hypothesis testing, the test statistic (e.g. *F*, *t*, *r*) with confidence intervals, effect sizes, degrees of freedom and *P* value noted<br>*Give P values as exact values whenever suitable.*
[x]	[ ]	For Bayesian analysis, information on the choice of priors and Markov chain Monte Carlo settings
[x]	[ ]	For hierarchical and complex designs, identification of the appropriate level for tests and full reporting of outcomes
[ ]	[x]	Estimates of effect sizes (e.g. Cohen's *d*, Pearson's *r*), indicating how they were calculated

Our web collection on statistics for biologists contains articles on many of the points above.

Software and code

Policy information about availability of computer code

Data collection

We used the UK Biobank (project ID: 52887). The data are available by request from UK Biobank but are not available freely due to data privacy laws. Full instructions on access of the data are here: https://biobank.ndph.ox.ac.uk/~bbdatan/Accessing_UKB_data_v2.3.pdf. We used no software for data collection. We confirm that our research complies with all ethnical regulations and is approved by UK Biobank (project ID: 52887) and was deemed not human subjects research by Harvard IRB (IRB16-2145) as defined by DHHS or FDA regulations; subjects are deidentified by the UK Biobank and we, the investigators, had no contact with the subjects.

Data analysis

Our code is accessible here: https://github.com/Deep-Learning-and-Aging. For genetics analyses, we used BOLT-LMM, BOLT-REML, and FUMA software. We coded the deep learning in Python using TensorFlow 2, Keras, and INNvestigate. Versions of each library can be found in our github repository and here: Python version 3.6.

beautifulsoup4==4.8.2
bioinfokit==0.8.8
bs4==0.0.1
efficientnet==1.1.0
gpuinfo==1.0.0a6
GPUtil==1.4.0
graphviz==0.13.2
hyperopt==0.1.2
imageio==2.5.0
innvestigate==1.0.8
ipdb==0.13.2
keract==4.0.0
Keras==2.3.1

Keras-Applications==1.0.8
Keras-Preprocessing==1.1.0
keras-vis==0.4.1
kerasplotlib==0.1.4
lifelines==0.25.6
lightgbm==2.3.1
matplotlib==3.3.3
matplotlib-venn==0.11.5
more-itertools==7.2.0
multiprocess==0.70.11.1
numpy==1.18.5
nvgpu==0.7.0
nvidia-ml-py==375.53.1
opencv-python==4.1.1.26
opt-einsum==3.2.1
pandas==0.25.3
pickleshare==0.7.5
Pillow==8.0.1
pydicom==1.3.0
scikit-image==0.14.2
scikit-learn==0.23.0
scipy==1.4.1
seaborn==0.9.0
six==1.12.0
sklearn==0.0
tensorflow-addons==0.10.0
tensorflow-estimator==2.2.0
tensorflow-gpu==2.2.0
tf-estimator-nightly==1.14.0.dev2019030115
tf-keras-vis==0.3.1
threadpoolctl==2.0.0
utils==1.0.1
virtualenv==15.1.0
xgboost==0.82

For manuscripts utilizing custom algorithms or software that are central to the research but not yet described in published literature, software must be made available to editors and reviewers. We strongly encourage code deposition in a community repository (e.g. GitHub). See the Nature Portfolio guidelines for submitting code & software for further information.

Data

Policy information about availability of data

All manuscripts must include a data availability statement. This statement should provide the following information, where applicable:

- Accession codes, unique identifiers, or web links for publicly available datasets
- A description of any restrictions on data availability
- For clinical datasets or third party data, please ensure that the statement adheres to our policy

The data are available by request from UK Biobank but are not available freely due to data privacy laws. The processed age predictions will be available at request from UK Biobank. The results can be interactively and extensively explored at https://www.multidimensionality-of-aging.net/, a website where we display and compare the performance and properties of the different biological age predictors we built. Select "Abdomen" as the aging dimension on the different pages to display the subset of the results relevant to this publication. The GWAS results (and summary statistics via FigShare) can be found here: AbdAge: https://fuma.ctglab.nl/browse/400 (via FigShare: 10.6084/m9.figshare.19361999 and https://figshare.com/articles/dataset/GWAS_Age_Abdomen_X_bgen_stats_gz/19361999), Liver Age: https://fuma.ctglab.nl/browse/401 (via FigShare: 10.6084/m9.figshare.19361972 and https://figshare.com/articles/dataset/GWAS_Age_AbdomenLiver_X_bgen_stats_gz/19361972) and Pancreas Age: https://fuma.ctglab.nl/browse/402 (via FigShare: 10.6084/m9.figshare.19361957 and https://figshare.com/articles/dataset/GWAS_Age_AbdomenPancreas_X_bgen_stats_gz/19361957).

Field-specific reporting

Please select the one below that is the best fit for your research. If you are not sure, read the appropriate sections before making your selection.

☒ Life sciences ☐ Behavioural & social sciences ☐ Ecological, evolutionary & environmental sciences

For a reference copy of the document with all sections, see nature.com/documents/nr-reporting-summary-flat.pdf

Life sciences study design

All studies must disclose on these points even when the disclosure is negative.

Sample size	We leveraged the full sample size provided by UK Biobank to conduct all analyses.
Data exclusions	We filtered out samples with poor data quality (missing data or corrupted images).
Replication	We used ten fold cross-validation to replicate.
Randomization	Each individual was randomly allocated to ten data folds for cross-validation.
Blinding	The investigators were blinded to the data collection process and did not participate in the allocation of groups or measurement of the cohort.

Reporting for specific materials, systems and methods

We require information from authors about some types of materials, experimental systems and methods used in many studies. Here, indicate whether each material, system or method listed is relevant to your study. If you are not sure if a list item applies to your research, read the appropriate section before selecting a response.

Materials & experimental systems

n/a	Involved in the study	
☒	☐	Antibodies
☒	☐	Eukaryotic cell lines
☒	☐	Palaeontology and archaeology
☒	☐	Animals and other organisms
☐	☒	Human research participants
☒	☐	Clinical data
☒	☐	Dual use research of concern

Methods

n/a	Involved in the study	
☒	☐	ChIP-seq
☒	☐	Flow cytometry
☒	☐	MRI-based neuroimaging

Human research participants

Policy information about studies involving human research participants

Population characteristics	The UKB consists of cohort data from 502,211 de-identified participants aged 37-74 at enrollment that started in 2006. Participants were 45-81 years of age when sampled for our analysis. The gender ratio is biased toward females (54.4% females). The population is White primarily (95%).
Recruitment	The participants are recruited by the UK Biobank team. The protocol is here: https://www.ukbiobank.ac.uk/media/gnkeyh2q/study-rationale.pdf
Ethics oversight	The Harvard Internal Review Board deemed this research as non human subjects research (IRB 16-2145). We confirm that our research complies with all ethnical regulations and is approved by UK Biobank (project ID: 52887) and was deemed not human subjects research by Harvard IRB (IRB16-2145) as defined by DHHS or FDA regulations; subjects are deidentified by the UK Biobank and we, the investigators, had no contact with the subjects.

Note that full information on the approval of the study protocol must also be provided in the manuscript.
