# Supplementary material for: Using deep learning to predict abdominal age from liver and pancreas magnetic resonance images
Source: Nat Commun. 2022 Apr 13;13:1979. doi: 10.1038/s41467-022-29525-9 (PMC9007982; doi:10.1038/s41467-022-29525-9)
Supplement: Supplementary file 3 — Description of Additional Supplementary Files [file 41467_2022_29525_MOESM3_ESM.pdf]

Title: Supplementary Data 1**:**
Description: Genome-wide association study summary statistics. All SNPs in LD to the main SNP for each locus are shown. All p-values are two sided and not corrected for multiple comparisons. SE: standard error.

Title: Supplementary Data 2:

Description: List of biomarkers by subcategories for the Biomarkers Wide Association Study [BWAS]

Title: Supplementary Data 3:

Description: Biomarkers most associated with accelerated aging for each abdominal aging dimension

Title: Supplementary Data 4:

Description: Biomarkers most associated with decelerated aging for each abdominal aging dimension

Title: Supplementary Data 5:

Description: List of clinical phenotypes by subcategories for the Clinical Phenotypes Wide Association Study [CWAS]

Title: Supplementary Data 6:

Description: Clinical phenotypes most associated with accelerated aging for each abdominal aging dimension

Title: Supplementary Data 7:

Description: Clinical phenotypes most associated with decelerated aging for each abdominal aging dimension

Title: Supplementary Data 8:

Description: List of diseases by subcategories for the Diseases Wide Association Study [DWAS]

Title: Supplementary Data 9:

Description: Diseases most associated with accelerated aging for each abdominal aging dimension

Title: Supplementary Data 10:

Description: Diseases most associated with decelerated aging for each abdominal aging dimension

Title: Supplementary Data 11:

Description: List of family history variables by subcategories for the Family History Phenotypes Wide Association Study [FWAS]

Title: Supplementary Data 12:

Description: Family history variables most associated with accelerated aging for each abdominal dimension

Title: Supplementary Data 13:

Description: Family history variables most associated with decelerated aging for each abdominal aging dimension

Title: Supplementary Data 14:

Description: List of environmental variables by subcategories for the Environment Wide Association Study [EWAS]

Title: Supplementary Data 15:

Description: Environmental variables most associated with accelerated aging for each abdominal aging dimension

Title: Supplementary Data 16:

Description: Environmental variables most associated with decelerated aging for each abdominal aging dimension

Title: Supplementary Data 17:

Description: List of socioeconomic variables by subcategories for the Socioeconomics Wide Association Study [SWAS]

Title: Supplementary Data 18:

Description: Socioeconomic variables most associated with accelerated aging for each abdominal aging dimension

Title: Supplementary Data 19:

Description: Socioeconomic variables most associated with decelerated aging for each abdominal aging dimension

Title: Supplementary Data 20:

Description: Exhaustive XWAS results - association between non-genetic factors and

accelerated aging in each abdominal dimension. All p-values are two sided and not corrected for multiple comparisons.

Title: Supplementary Data 21:

Description: Image sizes after resizing

Title: Supplementary Data 22:

Description: Hyperparameters tuning upstream of the cross-validation for images-based models

Title: Supplementary Data 23:

Description: Outer Cross-Validation with inner split pipeline

The values displayed are validation RMSE values. Lower values are associated with better hyperparameter tuning. When two values are displayed (value1/value2), the second value corresponds to the training RMSE. The architecture used was InceptionV3, with an initial learning rate of 0.001. The model was trained on the data folds 2-9, and validated on the data fold #0. The data fold #1 was set aside as the testing set and was not used.

Title: Supplementary Data 24:

Description: Hyperparameter space for scalar features-based models Bayesian optimization

Title: Supplementary Data 25:

Description: Nested Cross-Validation pipeline
